# First Metal-Free Synthesis of Tetracyclic Pyrido and Pyrazino Thienopyrimidinone Molecules

**DOI:** 10.3390/molecules23051159

**Published:** 2018-05-11

**Authors:** Mohammed Aounzou, Joana F. Campos, Mohammed Loubidi, Sabine Berteina-Raboin

**Affiliations:** Institut de Chimie Organique et Analytique, Université d’Orléans—Pôle de Chimie, UMR CNRS 7311, Rue de Chartres—BP 6759, 45067 Orléans CEDEX 2, France; mohammed.aounzou@etu.univ-orleans.fr (M.A.); joana-filomena.mimoso-silva-de-campos@univ-orleans.fr (J.F.C.); m.loubidi@gmail.com (M.L.)

**Keywords:** metal-free, thienopyrimidinone, tetracyclic compounds

## Abstract

We report herein a new metal free synthetic pathway to generate tetracyclic compounds from 3-aminothieno[3,2-*b*]pyridine-2-carboxylate. To enlarge the molecular diversity, we studied the Suzuki coupling of 9-chloro-6*H*-pyrido[1,2-*a*]pyrido[2′,3′:4,5]thieno[3,2-*d*]pyrimidin-6-one and several boronic acids were easily introduced.

## 1. Introduction

**Figure 1:**
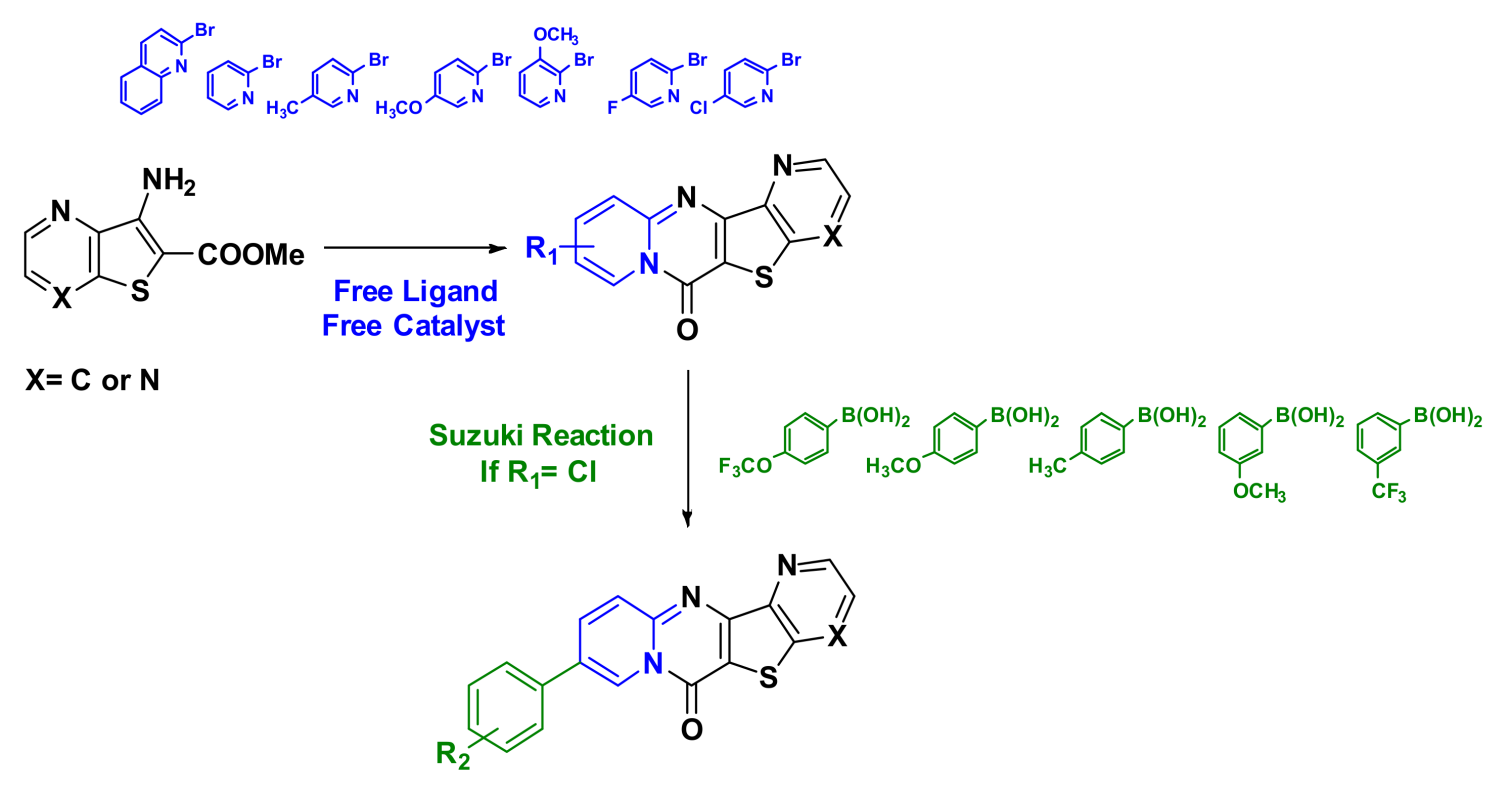
Metal free synthesis of several new tetracyclic compounds from 3-aminothieno[3,2-*b*]pyridine-2-carboxylate.

Angiogenesis, one of the critical processes that affect the growth and development of cancerous cells, is the generation of new blood vessels from the existing vasculature. It is the key factor in the advancement of various human diseases, including cancer, where it is essential for the growth, spread, and survival of tumors. Angiogenesis is a complex process regulated by multiple growth factors and cytokines. Among these factors, vascular endothelial growth factor (VEGF) is one of the most potent angiogenic factors involved in tumor growth. It stimulates endothelial cell proliferation, migration, and tube formation by binding to its two main receptor tyrosine kinases (RTKs) expressed on endothelial cells, VEGF receptor 1 (VEGFR-1) and VEGF receptor 2 (VEGFR-2) [1]. Thienopyridine derivatives have applications in many areas and have shown interesting biological activities in medicinal chemistry or agricultural chemistry [2,3,4]. Various thieno[3,2-*b*]pyridine, thieno[2,3-*d*]pyrimidine and thieno[3,2-*d*]pyrimidine skeletons have frequently attracted the interest of medicinal chemistry researchers due to their promising anticancer properties. They were shown to be inhibitors of the vascular endothelial growth factor receptor (VEGFR-2) related to angiogenesis and metastasis. These compounds are also involved in cyclin dependent kinase (CDK4) and many other anti-cancer therapies [5]. In this work, we describe the simple and efficient metal free synthesis of several new tetracyclic compounds from 3-aminothieno[3,2-*b*]pyridine-2-carboxylate (Figure 1) followed by suzuki cross-coupling to enlarge the molecular diversity. The advantages of using transition metal catalysts in synthesis are now widely known, however it is also necessary to take into account certain essential factors for efficient and environmentally friendly syntheses: cost, toxicity, and durability. Our team has focused its research activities on the development of greener methodologies and approaches. In recent years, several studies have been reported in the literature with the demonstration of alternative synthetic routes to obtain sulfur and nitrogen containing heterocycles [6,7]. As far as we know, there are only three descriptions of the synthesis of these tetracyclic structures in the literature, all of which reported moderate yields and involved a Buchwald–Hartwig C-N coupling [8,9,10,11,12] of substituted 3-bromobenzo[*b*]thiophenes with anilines and amino-pyridines [13], 3-aminobenzo[*b*]thiophenes with bromopyridines [14].

## 2. Results and Discussion

In 2010, this reaction was performed and described once from 3-aminothieno[3,2-*b*]pyridine-2-carboxylate and 2-bromopyridine [15]. The 3-aminothieno[3,2-*b*]pyridine-2-carboxylate **1** as starting material for palladium-catalyzed coupling by reaction on the 2-bromopyridine was prepared in excellent yield, reacting the 3-fluoropicolinonitrile with methyl thioglycolate in DMF using an excess of KOH (aq), following the known procedure [14]. The optimization of C-N Buchwald–Hartwig coupling of 3-amino-thieno[3,2-*b*]pyridine-2-carboxylate **1** with 2-bromopyridine was performed to obtain tetracyclic compounds in good yields (Table 1). These compounds are generated, after Palladium coupling, by intramolecular cyclization involving the nucleophilic attack of the pyridine nitrogen on the carbonyl of the ester group, described on the benzo[*b*]thiophene series [15].

**Scheme 1:**
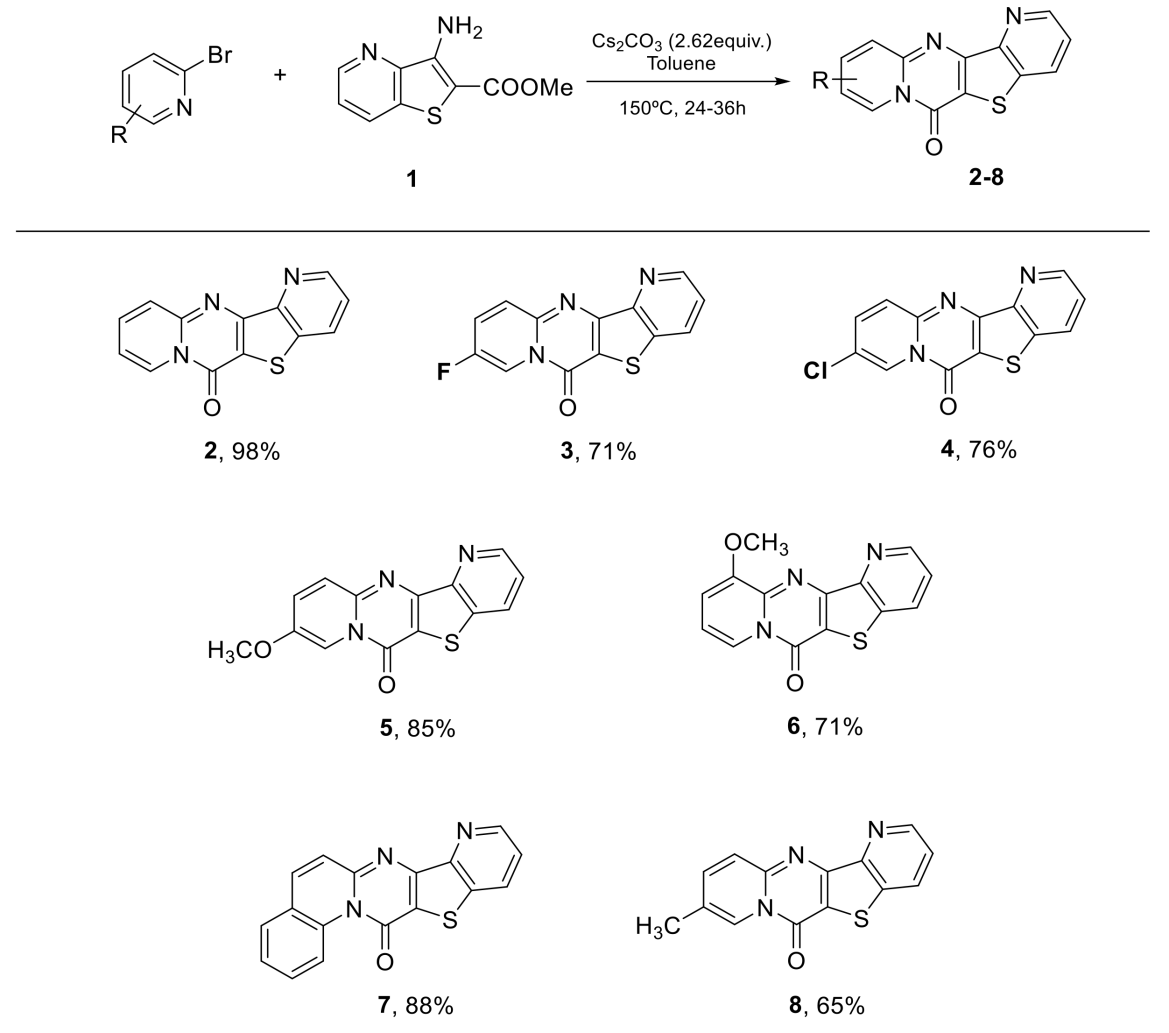
Synthesis of new tetracyclic compounds from 3-aminothieno[3,2-*b*]pyridine-2-carboxylate.

Our goal was to perform the Buchwald–Hartwig Reaction under the conditions reported in the literature [8,9,10,11,12], in order to optimize the yield and achieve a more environmentally benign process by decreasing the quantity of palladium and reagents. To generate the tetracyclic compound **2** we first used 2-bromopyridine and methyl 3-aminothieno[3,2-*b*]pyridine-2-carboxylate **1** in presence of Pd(OAc)_2_ and Xantphos as ligand in 1,4 dioxane at 120 °C for 24 h (entry 1, Table 1) to led to **2** in 67% yield using the conditions usually applied in our team to optimize cross-coupling methodologies [11,12]. We then used several less complex ligands and various solvents. Unfortunately, with PPh_3_ and 10-phenanthroline or without ligand a decrease in the yields was obtained (entries 2–4 and 6–7, Table 1). When the temperature was increased, the reaction could be performed without any ligand (entry 5 versus entry 9). When K_3_PO_4_ was used instead of Cs_2_CO_3_ no product was obtained (entry 10). The same result was found when we used a mixture of toluene/EtOH: 2/1 instead of toluene (entry 8). Degradation was observed under microwave irradiation (MW) at 150 °C in a sealed reaction vessel (entry 11). This reaction was carried out in a Biotage Initiator microwave synthesis instrument, the temperature was measured by IR-sensor and was maintained during the experiment. With Palladium(II) trifluoroacetate without ligand, a reasonable yield of 61% was obtained at 120 °C (entry 12) but was unsuccessful with Pd(dppf)Cl_2_·DCM (entry 13). We finally explored the reaction without Palladium catalyst and to our surprise, in view of the methods already published using exclusively palladium catalyst, the expected product was obtained in quasi quantitative yield using only Cs_2_CO_3_ and toluene as solvent (entry 14). With these optimized conditions, the scope and limitation of the reaction were examined (Scheme 1).

**Scheme 2:**
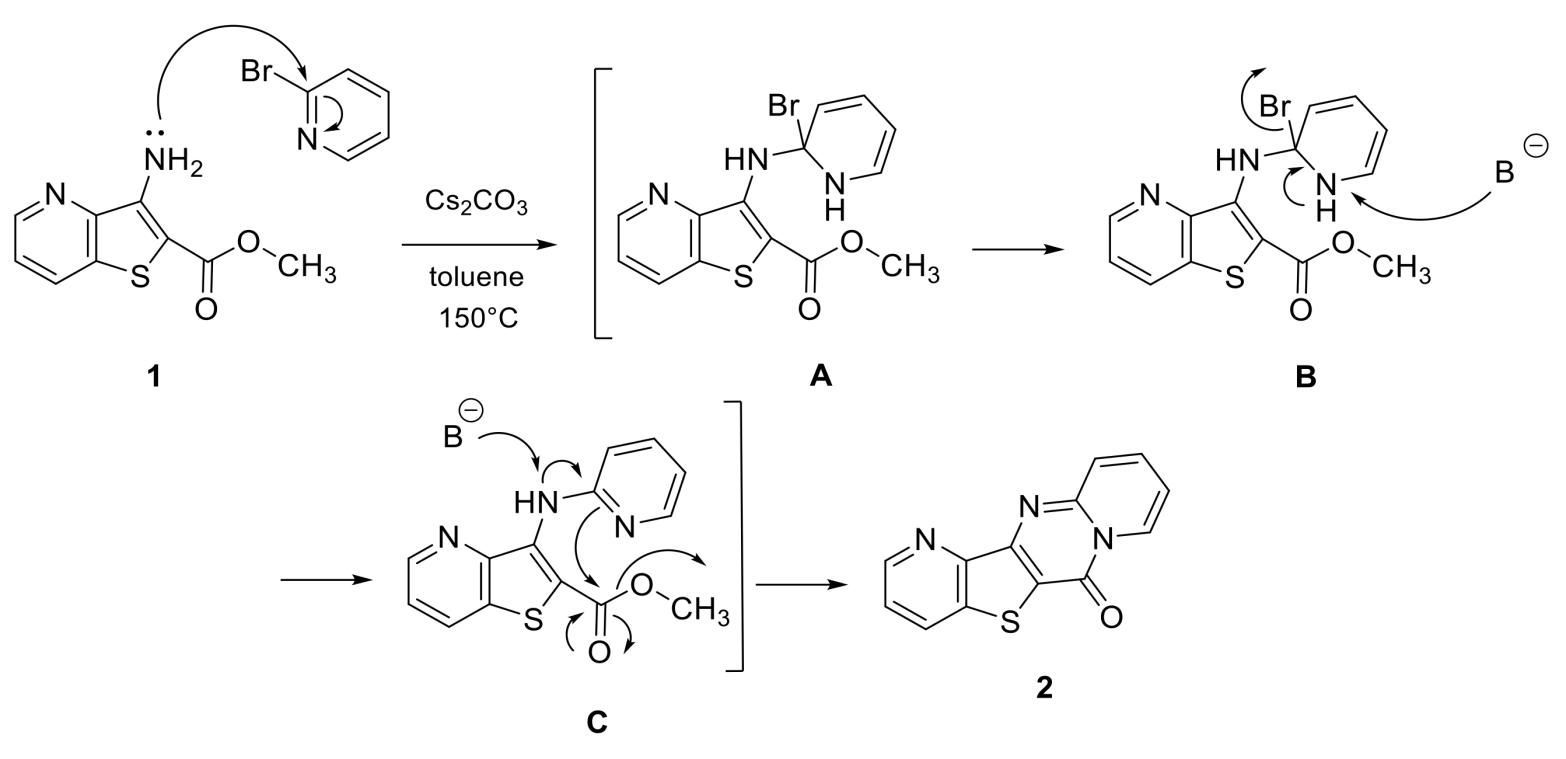
Plausible mechanism.

Different 2-bromo pyridines were then used for the reaction. Several 6*H*-pyrido[1,2-*a*]pyrido [2′,3′:4,5]thieno[3,2-*d*]pyrimidin-6-ones were obtained in good to excellent yield. Some bromopyridine derivatives required more time to achieve total consumption of the starting material. In contrast to already published results, this reaction did not proceed via C-N Buchwald–Hartwig Cross Coupling. The mechanism is represented in Scheme 2: an electrophilic attack of amine followed by an elimination of HBr in presence of Cs_2_CO_3_ gave the intermediate **C** (Scheme 2) which underwent an intramolecular cyclization process.

**Scheme 3:**
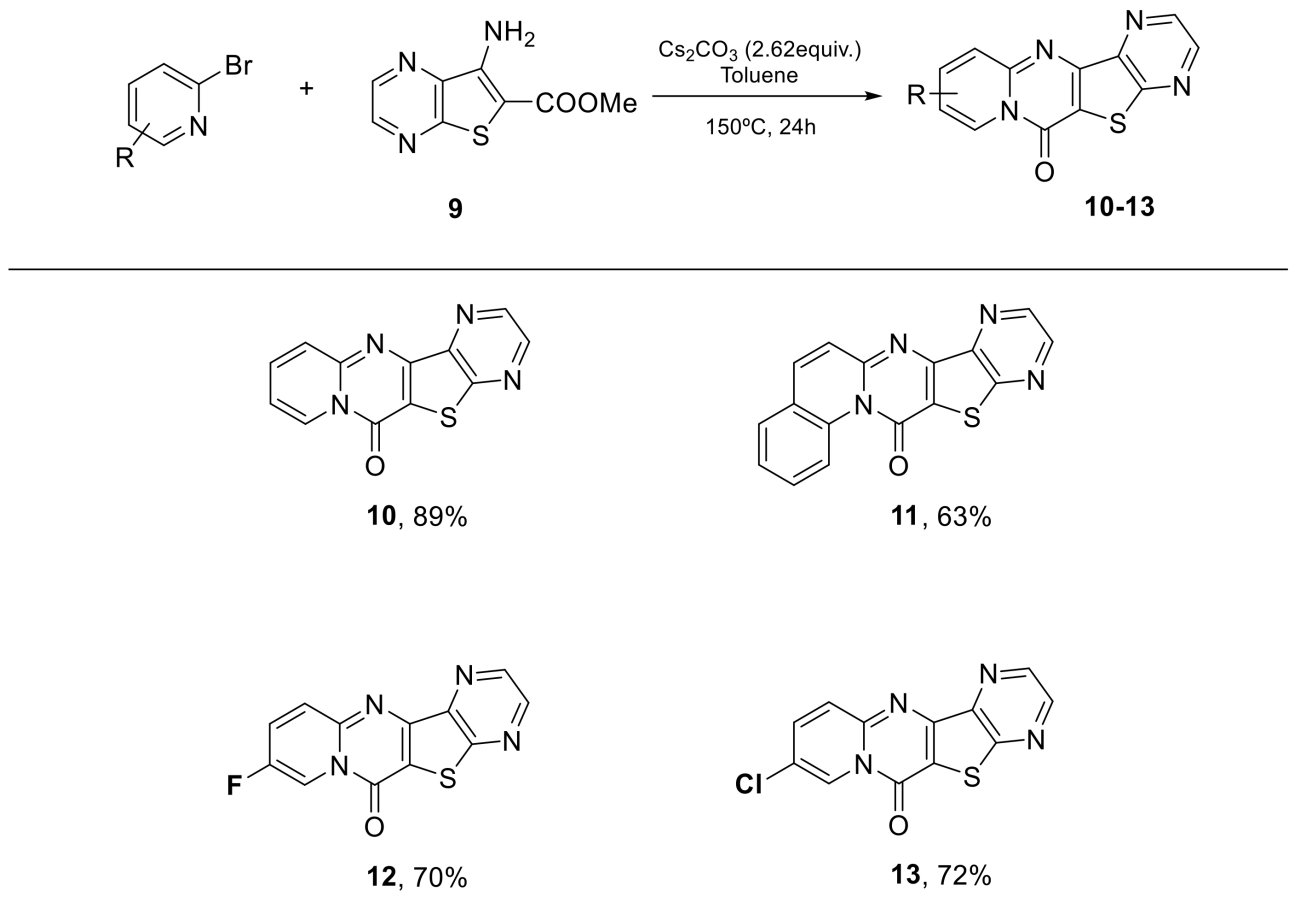
Synthesis of new tetracyclic compounds from methyl 7-aminothieno[2,3-*b*]pyrazine-6-carboxylate.

The conditions found were tested on pyrazine scaffold. From methyl 7-aminothieno[2,3-*b*]pyrazine-6-carboxylate (**9**), several 6*H*-pyrido[1,2-*a*]pyrido[2′,3′:4,5]thieno[3,2-*d*]pyrimidinones were synthesized in moderate to good yields that demonstrated the generality of this method. Based on the latest results of the team, as we expected, the pyrazine showed lower yields than the pyridine (Scheme 3).

In the second phase of this work, we studied the Suzuki Reaction of 6*H*-pyrido[1,2-*a*]pyrido [2′,3′:4,5]thieno[3,2-*d*]pyrimidin-6-one **4** with *p*-tolylboronic acid (Table 2).

**Scheme 4:**
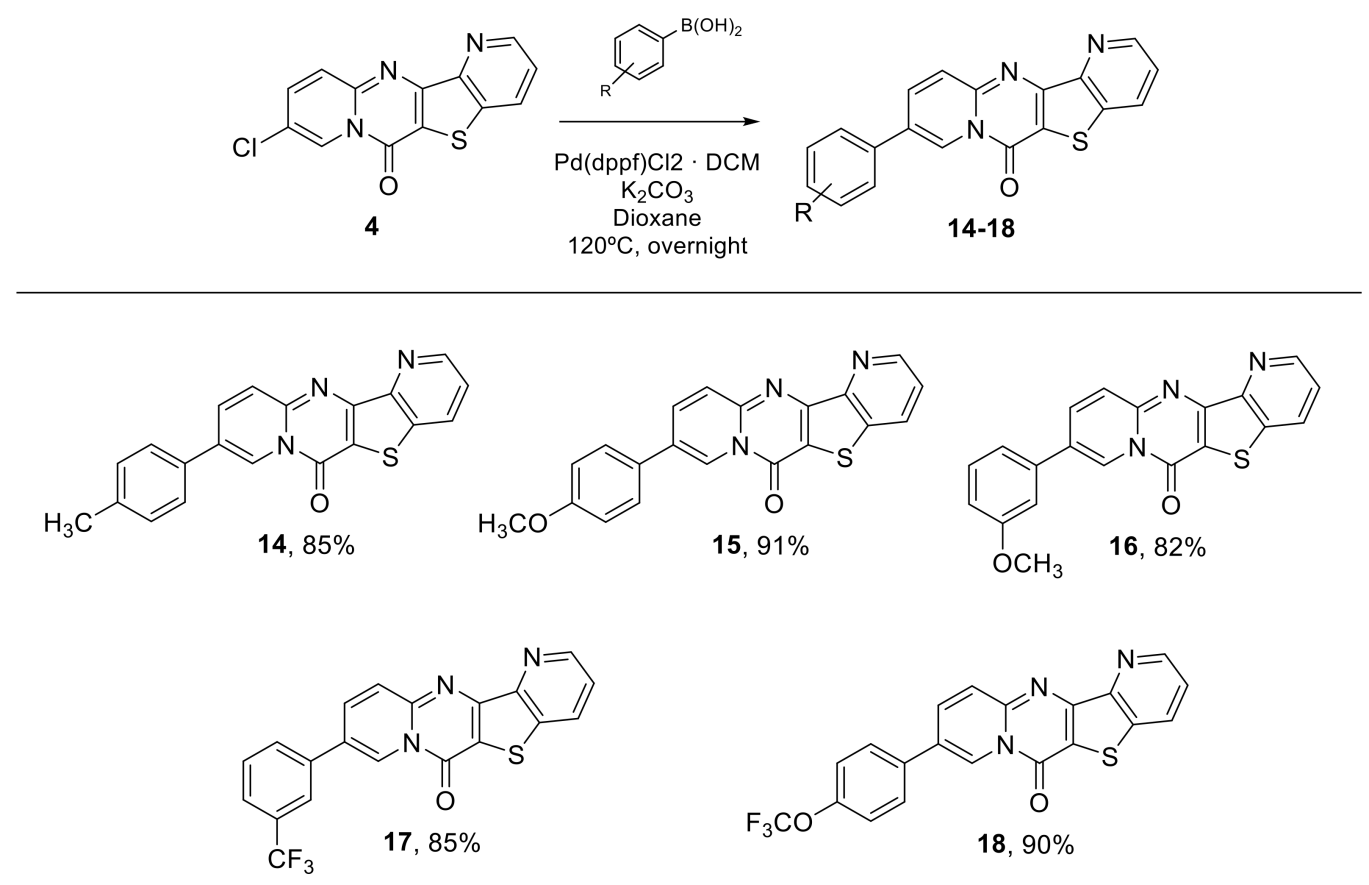
Synthesis of 6*H*-pyrido[1,2-*a*]pyrido[2′,3′:4,5]thieno[3,2-*d*]pyrimidin-6-one from compound **4**.

The experimental conditions used were those previously reported by our team [12,16,17,18,19,20]. The best result, 85% yield, was obtained with K_2_CO_3_ as base and Pd(dppf)Cl_2_·DCM complex as catalyst. The use of Na_2_CO_3_ decreased the yield (entry 2, Table 2) and changing the catalyst led to a mixture of various compounds. With our optimized conditions, the scope and limitation of the reaction were examined (Table 2 and Scheme 4). Several boronic acids were used for the reaction. Using Pd(dppf)Cl_2_·DCM (10 mol%), K_2_CO_3_ (2 equiv.) in dioxane at 120 °C for 24 h, the various 6*H*-pyrido[1,2-*a*]pyrido [2′,3′:4,5]thieno[3,2-*d*]pyrimidin-6-ones were obtained in excellent yield (Scheme 4).

## 3. Materials and Methods

### 3.1. General Methods

All reagents were purchased from commercial suppliers and were used without further purification except for DMF, which was stored under argon and activated molecular sieves. The reactions were monitored by thin-layer chromatography (TLC) analysis using silica gel (60 F254) plates. Compounds were visualized by UV irradiation. Flash column chromatography was performed on silica gel 60 (230–400 mesh, 0.040–0.063 mm). Melting points (m.p. [°C]) were taken on samples in open capillary tubes and are uncorrected. ^1^H and ^13^C NMR spectra were recorded on a Bruker DPX 250 (13C, 62.9 MHz) (Bruker, Wissembourg, France), Bruker Avance II 250.13 (13C, 63 MHz), Bruker Avance 400.13 (13C, 101 MHz) (Bruker, Wissembourg, France), or on a Bruker Avance III HD nanobay 400.13 (13C, 101 MHz) (Bruker, Wissembourg, France). Chemical shifts are given in parts per million from tetramethylsilane (TMS) as internal standard. The following abbreviations are used for the proton spectra multiplicities: b: broad, s: singlet, d: doublet, t: triplet, q: quartet, p: pentuplet, m: multiplet. Coupling constants (*J*) are reported in hertz (Hz). Multiplicities were determined by the DEPT 135 sequence. Attributions of protons and carbons were made with the help of HSQC and HMBC 2D NMRs. Eudesmane numbering of carbons was used instead of the IUPAC numbering. Microwaves-assisted reactions were carried out in a Biotage Initiator microwave synthesis instrument and temperatures were measured by IR-sensor (Biotage, Uppsala, Sweden), High-resolution mass spectra (HRMS) were performed on a Maxis UHR-q-TOF mass spectrometer Bruker 4G (Bruker, Wissembourg, France), with an electrospray ionisation (ESI) mode.

### 3.2. General Methods

General Procedure for Synthesis of 6*H*-pyrido[1,2-*a*]pyrido[2′,3′:4,5]thieno[3,2-*d*]pyrimidin-6-ones (**2**–**8** and **10**–**13**). A solution of compound **1** or **9** (100 mg; 0.480 mmol), 2-bromo pyridine (2 equiv., 0.960 mmol) and Cs_2_CO_3_ (2.62 equiv., 1,258 mmol) was heated at 150 °C in dry toluene (2 mL) for 24–36 h. The reaction was followed by TLC. After completion, the mixture was concentrated under vacuum. The solid obtained was submitted to a column chromatography. The increase of polarity in solvent gradient was made from neat petroleum ether to mixture of AcOEt/petroleum ether (9:1). ^1^H NMR and ^13^C NMR of compounds **2**–**8**, **10**–**18** are available in Supplementary Materials.

*6H-Pyrido[1,2-a]pyrido[2′,3′:4,5]thieno[3,2-d]pyrimidin-6-one* (**2**) [15]: Yellow solid (121 mg, 98%), m.p. 280–282 °C. ^1^H NMR (400 MHz, CDCl_3_) δ 7.17 (td, *J* = 1.4, 7.2 Hz, 1H), 7.53 (dd, *J* = 4.5, 8.3 Hz, 1H), 7.74 (ddd, *J* = 1.6, 6.6, 8.3 Hz, 1H), 7.98 (d, *J* = 9.0 Hz, 1H), 8.30 (dd, *J* = 1.4, 8.3 Hz, 1H), 8.93 (dd, *J* = 1.4, 4.5 Hz, 1H), 9.11 (d, *J* = 7.2 Hz, 1H) ppm. ^13^C NMR (101 MHz, CDCl_3_) δ 115.1, 116.4, 123.4, 126.5, 127.3, 131.8, 135.0, 137.5, 148.5, 149.7, 150.0, 152.7, 155.1 ppm.

*9-Fluoro-6H-pyrido[1,2-a]pyrido[2′,3′:4,5]thieno[3,2-d]pyrimidin-6-one* (**3**): Yellow solid (92 mg, 71%), m.p. 334–336 °C. ^1^H NMR (400 MHz, CDCl_3_) δ 7.57 (dd, *J* = 8.3, 4.5 Hz, 1H), 7.68 (ddd, *J* = 2.8, 6.5, 9.6 Hz, 1H), 8.02 (dd, *J* = 5.3, 9.9 Hz, 1H), 8.33 (dd, *J* = 1.4, 8.3 Hz, 1H), 8.95 (dd, *J* = 1.4, 4.5 Hz, 1H), 9.03 (dd, *J* = 2.8, 4.8 Hz, 1H) ppm. ^13^C NMR (101 MHz, CDCl_3_) δ 112.7, 116.6, 123.6, 128.1, 129.2, 131.9, 137.5, 148.7, 149.6, 152.5, 152.8, 154.6, 155.2 ppm. HRMS: calcd for C_13_H_7_FN_3_OS [M + H]^+^ 272.0288, found 272.0289.

*9-Chloro-6H-pyrido[1,2-a]pyrido[2′,3′:4,5]thieno[3,2-d]pyrimidin-6-one* (**4**): Yellow solid (105 mg, 76%), m.p. 323–325 °C. ^1^H NMR (400 MHz, CDCl_3_) δ 7.57 (dd, *J* = 4.3, 8.3 Hz, 1H), 7.68 (d, *J* = 9.6 Hz, 1H), 7.95 (d, *J* = 9.6 Hz, 1H), 8.34 (d, *J* = 8.3 Hz, 1H), 8.96 (d, *J* = 3.6 Hz, 1H), 9.16 (s, 1H) ppm. ^13^C NMR (101 MHz, CDCl_3_) δ 123.6, 123.9, 124.2, 128.2, 131.9, 136.4, 137.6, 148.4, 148.8, 149.5, 152.5, 154.2, 162.6 ppm. HRMS: calcd for C_13_H_7_ClN_3_OS [M + H]^+^ 287.9993, found 287.9994.

*9-Methoxy-6H-pyrido[1,2-a]pyrido[2′,3′:4,5]thieno[3,2-d]pyrimidin-6-one* (**5**): Yellow solid (116 mg, 85%), m.p. 361–363 °C. ^1^H NMR (250 MHz, CDCl_3_) δ 3.99 (s, 3H), 7.50–7.58 (m, 2H), 7.90–7.99 (m, 1H), 8.31 (dd, *J* = 1.5, 8.3 Hz, 1H), 8.61 (d, *J* = 2.6 Hz, 1H), 8.94 (dd, *J* = 1.4, 4.5 Hz, 1H) ppm. ^13^C NMR (101 MHz, CDCl_3_) δ 56.5, 105.7, 115.9, 123.1, 127.8, 130.9, 131.7, 137.1, 147.2, 148.3, 149.8, 150.6, 151.8, 154.7 ppm. HRMS: calcd for C_14_H_10_N_3_O_2_S [M + H]^+^ 284.0488, found 284.0491.

*11-Methoxy-6H-pyrido[1,2-a]pyrido[2′,3′:4,5]thieno[3,2-d]pyrimidin-6-one* (**6**): Yellow solid (96 mg, 71%), m.p. 347–349 °C. ^1^H NMR (250 MHz, CDCl_3_) δ 4.07 (s, 3H), 6.98 (dd, *J* = 1.4, 7.7 Hz, 1H), 7.07 (t, *J* = 7.4 Hz, 1H), 7.51 (dd, *J* = 4.5, 8.3 Hz, 1H), 8.28 (dd, *J* = 1.4, 8.3 Hz, 1H), 8.73 (dd, *J* = 1.4, 7.2 Hz, 1H), 8.93 (dd, *J* = 1.4, 4.5 Hz, 1H) ppm. ^13^C NMR (101 MHz, CDCl_3_) δ 53.4, 122.3, 125.1, 125.3, 127.1, 128.2, 129.6, 131.7, 135.7, 148.6, 149.4, 149.8, 150.7, 159.8 ppm. HRMS: calcd for C_14_H_10_N_3_O_2_S [M + H]^+^ 284.0488, found 284.0488.

*13H-Pyrido[2′’,3′’:4′,5′]thieno[3′,2′:4,5]pyrimido[1,2-a]quinolin-13-one* (**7**): Yellow solid (128 mg, 88%), m.p. 282–284 °C. ^1^H NMR (250 MHz, CDCl_3_) δ 7.51–7.60 (m, 2H), 7.65–7.74 (m, 3H), 7.80 (d, *J* = 9.3 Hz, 1H), 8.32 (d, *J* = 7.3 Hz, 1H), 8.94 (d, *J* = 4.6 Hz, 1H), 9.94 (d, *J* = 8.7 Hz, 1H) ppm. ^13^C NMR (101 MHz, CDCl_3_) δ 122.2, 122.3, 122.9, 125.1, 125.3, 127.1, 128.2, 129.6, 131.7, 135.6, 135.7, 137.3, 148.6, 149.5, 149.8, 150.7, 159.8 ppm. HRMS: calcd for C_17_H_10_N_3_OS [M + H]^+^ 304.0539, found 304.0541.

*9-Methyl-6H-pyrido[1,2-a]pyrido[2′,3′:4,5]thieno[3,2-d]pyrimidin-6-one* (**8**): Yellow solid (83 mg, 65%), m.p. 277–279 °C. ^1^H NMR (400 MHz, CDCl_3_) δ 2.49 (s, 3H), 7.54 (dd, *J* = 4.5, 8.3 Hz, 1H), 7.62 (dd, *J* = 2.1, 9.2 Hz, 1H), 7.94 (d, *J* = 9.2 Hz, 1H), 8.31 (dd, *J* = 1.5, 8.2 Hz, 1H), 8.94–8.95 (m, 2H) ppm. ^13^C NMR (101 MHz, CDCl_3_) δ 18.7, 116.2, 123.3, 123.8, 125.4, 126.7, 131.8, 137.5, 138.2, 148.5, 149.1, 149.9, 152.6, 155.0 ppm. HRMS: calcd for C_14_H_10_N_3_OS [M + H]^+^ 268.0539, found 268.0541.

*6H-Pyrazino[2′,3′:4,5]thieno[3,2-d]pyrido[1,2-a]pyrimidin-6-one* (**10**): Yellow solid (108mg, 89%), m.p. 294–296 °C. ^1^H NMR (400 MHz, CDCl_3_) δ 7.26–7.30 (m, 1H), 7.83–7.86 (m, 1H), 8.03 (d, *J* = 9.1 Hz, 1H), 8.83 (s, 1H), 8.95 (s, 1H), 9.20 (d, *J* = 7.3 Hz, 1H) ppm. ^13^C NMR (100.6 MHz, CDCl_3_) δ 115.4, 116.1, 126.6, 127.1, 135.4, 142.8, 144.2, 144.9, 150.2, 150.3, 154.7, 159.0 ppm. HRMS: calcd for C_12_H_7_N_4_OS [M + H]^+^ 255.0335, found 255.0334.

*13H-Pyrazino[2′’,3′’:4′,5′]thieno[3′,2′:4,5]pyrimido[1,2-a]quinolin-13-one* (**11**): Yellow solid (92mg, 63%), m.p. 180–182 °C. ^1^H NMR (400 MHz, CDCl_3_) δ 7.60–64 (m, 1H). 7.70 (d, *J* = 9.4 Hz, 1H), 7.74–7.78 (m, 2H), 7.86 (d, *J* = 9.4 Hz, 1H), 8.78 (d, *J* = 2.3 Hz, 1H), 8.91 (d, *J* = 2.3 Hz, 1H), 9.96 (d, *J* = 8.9 Hz, 1H) ppm. ^13^C NMR (100.6 MHz, CDCl_3_) δ 115.6, 122.3, 125.0, 125.1, 127.4, 127.9, 128.3, 129.9, 130.7, 135.5, 136.3, 140.9, 142.9, 144.5, 159.5, 183.6 ppm. HRMS: calcd for C_16_H_9_N_4_OS [M + H]^+^ 305.0492, found 305.0492.

*9-Fluoro-6H-pyrazino[2′,3′:4,5]thieno[3,2-d]pyrido[1,2-a]pyrimidin-6-one* (**12**): Yellow solid (91 mg, 70%), m.p. 321–323 °C. ^1^H NMR (400 MHz, CDCl_3_) δ 7.71–7.76 (m, 1H), 8.00–8.04 (m, 1H), 8.80 (d, *J* = 2.3 Hz, 1H), 8.92 (d, *J* = 2.3 Hz, 1H), 9.06–9.08 (m, 1H) ppm. ^13^C NMR (100.6 MHz, CDCl_3_) δ 112.9, 128.2, 128.5, 128.9, 129.0, 142.9, 144.0, 145.0 ppm. HRMS: calcd for C_12_H_6_FN_4_OS [M + H]^+^ 273.0241, found 273.0242.

*9-Chloro-6H-pyrazino[2′,3′:4,5]thieno[3,2-d]pyrido[1,2-a]pyrimidin-6-one* (**13**): Yellow solid (99 mg, 72%), m.p. 342–344 °C. ^1^H NMR (400 MHz, CDCl_3_) δ 7.73 (dd, *J* = 9.6, 2.3 Hz, 1H), 7.94 (d, *J* = 9.6 Hz, 1H), 8.81 (d, *J* = 2.3 Hz, 1H), 8.92 (d, *J* = 2.3 Hz, 1H), 9.18 (d, *J* = 1.8 Hz, 1H) ppm. ^13^C NMR (100.6 MHz, CDCl_3_) δ 80.0, 124.2, 128.0, 136.8, 143.0, 145.1 ppm. HRMS: calcd for C_12_H_6_ClN_4_OS [M + H]^+^ 288.9945, found 288.9945.

General Procedure for Synthesis of 9-phenyl-6*H*-pyrido[1,2-*a*]pyrido[2′,3′:4,5]thieno[3,2-*d*]pyrimidin-6-one (**14**–**18**). A solution of compound **4** (100 mg; 0.348 mmol), boronic acid (2 equiv., 0.696 mmol), Pd(dppf)Cl_2_·DCM (10 mol%) and K_2_CO_3_ (2 equiv., 0.696 mmol) was heated at 120 °C in dioxane (2mL), overnight. After completion, the mixture was concentrated under vacuum. The solid obtained was submitted to a column chromatography. The increase of polarity in solvent gradient was made from neat petroleum ether to mixture of AcOEt/petroleum ether (6:4).

*9-(p-Tolyl)-6H-pyrido[1,2-a]pyrido[2′,3′:4,5]thieno[3,2-d]pyrimidin-6-one* (**14**): white solid (101 mg, 85%), m.p. 259 – 261 °C. ^1^H NMR (400 MHz, CDCl_3_) δ 2.44 (s, 3H), 7.34 (d, *J* = 7.7 Hz, 2H), 7.54–7.63 (m, 3H), 8.05 (d, *J* = 4.0 Hz, 2H), 8.33 (d, *J* = 8.1 Hz, 1H), 8.96 (d, *J* = 3.7 Hz, 1H), 9.32 (s, 1H) ppm. ^13^C NMR (101 MHz, CDCl_3_) δ 21.2, 115.8, 116.2, 122.8, 123.3, 126.8, 127.1, 128.8, 129.1, 130.1, 131.7, 132.6, 135.2, 137.4, 139.0, 148.4, 149.0, 149.7, 152.4, 155.0 ppm. HRMS: calcd for C_20_H_14_N_3_OS [M + H]^+^ 344.0852, found 344.0851.

*9-(4-Methoxyphenyl)-6H-pyrido[1,2-a]pyrido[2′,3′:4,5]thieno[3,2-d]pyrimidin-6-one* (**15**): white solid (113 mg, 91%), m.p. 274–276 °C. ^1^H NMR (400 MHz, CDCl_3_) δ 3.91 (s, 3H), 7.05–7.07 (m, 2H), 7.54–7.57 (m, 1H), 7.63–7.66 (m, 2H), 8.04 (dd, *J* = 2.0, 9.3 Hz, 2H), 8.33 (dd, *J* = 1.4, 8.2 Hz, 1H), 8.96 (dd, *J* = 1.5, 4.5 Hz, 1H), 9.29 (dd, *J* = 0.9, 2.0 Hz, 1H) ppm. ^13^C NMR (101 MHz, CDCl_3_) δ 55.5, 112.7, 114.2, 116.3, 119.3, 123.3, 123.3, 127.1, 128.9, 130.5, 131.7, 135.2, 136.9, 137.4, 148.4, 149.0, 149.6, 152.4, 155.0, 160.3 ppm. HRMS: calcd for C_20_H_14_N_3_O_2_S [M + H]^+^ 360.0801, found 360.0797.

*9-(3-Methoxyphenyl)-6H-pyrido[1,2-a]pyrido[2′,3′:4,5]thieno[3,2-d]pyrimidin-6-one* (**16**): white solid (102 mg, 82%), m.p. 278–280 °C. ^1^H NMR (400 MHz, CDCl_3_) δ 3.96 (s, 3H), 7.04–7.07 (m, 1H), 7.26–7.33 (m, 2H), 7.50 (t, *J* = 8.0 Hz, 1H), 7.61 (dd, *J* = 4.5, 8.2 Hz, 1H), 8.09–8.11 (m, 2H), 8.38 (dd, *J* = 1.5, 8.1 Hz, 1H), 9.01 (dd, *J* = 1.4, 4.4 Hz, 1H), 9.37–9.38 (m, 1H) ppm. ^13^C NMR (101 MHz, CDCl_3_) δ 55.5, 112.7, 114.2, 116.3, 119.3, 123.3, 123.3, 127.1, 128.9, 130.5, 131.7, 135.2, 136.9, 137.4, 148.4, 149.0, 149.6, 152.4, 155.0, 160.3 ppm. HRMS: calcd for C_20_H_14_N_3_O_2_S [M + H]^+^ 360.0801, found 360.0805.

*9-(3-(Trifluoromethyl)phenyl)-6H-pyrido[1,2-a]pyrido[2′,3′:4,5]thieno[3,2-d]pyrimidin-6-one* (**17**): white solid (118 mg, 85%), m.p. 265–267 °C. ^1^H NMR (400 MHz, CDCl_3_) δ 7.56 (dd, *J* = 4.5, 8.2 Hz, 1H), 7.67 (t, *J* = 7.7 Hz, 1H), 7.73 (d, *J* = 7.8 Hz, 1H), 7.89 (d, *J* = 7.7 Hz, 1H), 7.93 (s, 1H), 8.01 (dd, *J* = 2.1, 9.3 Hz, 1H), 8.09 (d, *J* = 9.3 Hz, 1H), 8.33 (dd, *J* = 1.5, 8.3 Hz, 1H), 8.95 (dd, *J* = 1.5, 4.5 Hz, 1H), 9.33 (d, *J* = 2.1 Hz, 1H) ppm. ^13^C NMR (101 MHz, CDCl_3_) δ 29.7, 116.6, 119.7, 122.4, 123.4, 123.8, 125.2, 125.6, 127.6, 130.0, 130.2, 131.7, 134.5, 136.5, 137.5, 148.6, 148.9, 149.5, 152.5, 154.9 ppm. HRMS: calcd for C_20_H_11_F_3_N_3_OS [M + H]^+^ 398.0569, found 398.0567.

*9-(4-(Trifluoromethoxy)phenyl)-6H-pyrido[1,2-a]pyrido[2′,3′:4,5]thieno[3,2-d]pyrimidin-6-one* (**18**): white solid (129 mg, 90%), m.p. 272–274 °C. ^1^H NMR (250 MHz, CDCl_3_) δ 7.37 (d, *J* = 8.3 Hz, 2H), 7.54 (dd, *J* = 4.5, 8.3 Hz, 1H), 7.71 (d, *J* = 8.6 Hz, 2H), 7.97 (dd, *J* = 2.2, 9.3 Hz, 1H), 8.06 (d, *J* = 9.3 Hz, 1H), 8.31 (dd, *J* = 1.4, 8.1 Hz, 1H), 8.94 (dd, *J* = 1.4, 4.3 Hz, 1H), 9.28 (d, *J* = 2.1 Hz, 1H) ppm. ^13^C NMR (101 MHz, CDCl_3_) δ 116.5, 116.6, 119.1, 121.8, 123.3, 123.4, 127.4, 127.6, 128.4, 131.7, 134.2, 134.6, 137.4, 148.5, 148.9, 149.5, 149.7, 149.7, 152.4, 154.9 ppm. HRMS: calcd for C_20_H_11_F_3_N_3_O_2_S [M + H]^+^ 414.0519, found 414.0518.

## 4. Conclusions

In summary, we have developed the first efficient method to obtain various new tetracyclic compounds from 3-aminothieno[3,2-*b*]pyridine-2-carboxylate in toluene without using ligand and catalyst. This procedure could be applied to various similar heterocycles to generate various biologically active compounds in an environmentally sound manner. In our case, to show the possible diversity we introduced several boronic acids by Suzuki reaction of 9-chloro-6*H*-pyrido[1,2-*a*]pyrido[2′,3′:4,5]thieno[3,2-*d*]pyrimidin-6-one in excellent yields.

## Figures and Tables

**Table 1:**
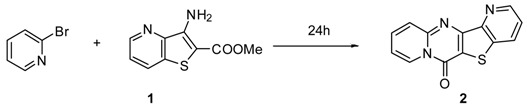
Optimization of conditions to generate tetracyclic compound **2**.

Entry	[Cat] (mol %)	Solvent	Ligand (mol %)	T (°C)	Base 2.62 equiv	Yield (%) ^[a]^
1	Pd(OAc)_2_ (3)	Dioxane	Xantphos (4)	120	Cs_2_CO_3_	67
2	Pd(OAc)_2_ (3)	Dioxane	PPh_3_ (6)	120	Cs_2_CO_3_	33
3	Pd(OAc)_2_ (5)	Dioxane	10-phenanthroline (10)	120	Cs_2_CO_3_	18
4	Pd(OAc)_2_ (3)	Dioxane	-	120	Cs_2_CO_3_	14
5	Pd(OAc)_2_ (3)	Toluene	-	120	Cs_2_CO_3_	^[b]^
6	Pd(OAc)_2_ (3)	Toluene	PPh_3_ (6)	120	Cs_2_CO_3_	8
7	Pd(OAc)_2_ (3)	Toluene	PPh_3_ (6)	150	Cs_2_CO_3_	10
8	Pd(OAc)_2_ (3)	Toluene/EtOH (2:1)	-	120	Cs_2_CO_3_	-
9	Pd(OAc)_2_ (3)	Toluene	-	150	Cs_2_CO_3_	41
10	Pd(OAc)_2_ (3)	Toluene	-	150	K_3_PO_4_	-
11	Pd(OAc)_2_ (3)	DMA	-	150	Cs_2_CO_3_	^[c]^
12	Pd(TFA)_2_ (5)	Toluene	-	120	Cs_2_CO_3_	61
13	Pd(dppf)Cl_2_·DCM (3)	Toluene	-	120	Cs_2_CO_3_	-
14	-	Toluene	-	150	Cs_2_CO_3_	98

^[a]^ Isolated yield after column chromatography. ^[b]^ 120 °C, t = 10 min. in MW 100% of starting material was recovered. ^[c]^ 150 °C, t = 1 h, MW degradation in mixture.

**Table 2:**
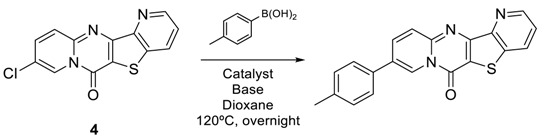
Suzuki reaction optimization in dioxane.

Entry	[Cat]	Base	% ^[a]^
1	Pd(dppf)Cl_2_·DCM	K_2_CO_3_	85
2	Pd(dppf)Cl_2_·DCM	Na_2_CO_3_	57
3	Pd(PPh_3_)_4_	Na_2_CO_3_	^[b]^

^[a]^ Isolated yield after column chromatography; ^[b]^ mixture of products.

## Supplementary Materials

The following are available online: ^1^H NMR and ^13^C NMR of compounds 2–8, 10–18.
