# Supplementary material for: First Metal-Free Synthesis of Tetracyclic Pyrido and Pyrazino Thienopyrimidinone Molecules
Source: Molecules. 2018 May 11;23(5):1159. doi: 10.3390/molecules23051159 (PMC6100531; doi:10.3390/molecules23051159)
Supplement: Supplementary file 1 [file molecules-23-01159-s001.pdf]

molecules

Article

First Metal-Free Synthesis of Tetracyclic Pyrido and Pyrazino Thienopyrimidinone Molecules

Mohammed Aounzou, Joana F. Campos, Mohammed Loubidi and Sabine Berteina-Raboin *

Institut de Chimie Organique et Analytique, Université d'Orléans - Pôle de chimie, UMR CNRS 7311, Rue de Chartres - BP 6759, 45067, Orléans Cedex 2, France

* Correspondence: sabine.berteina-raboin@univ-orleans.fr; Tel.: +33-238-494-856

6*H*-pyrido[1,2-*a*]pyrido[2',3':4,5]thieno[3,2-*d*]pyrimidin-6-one (2)

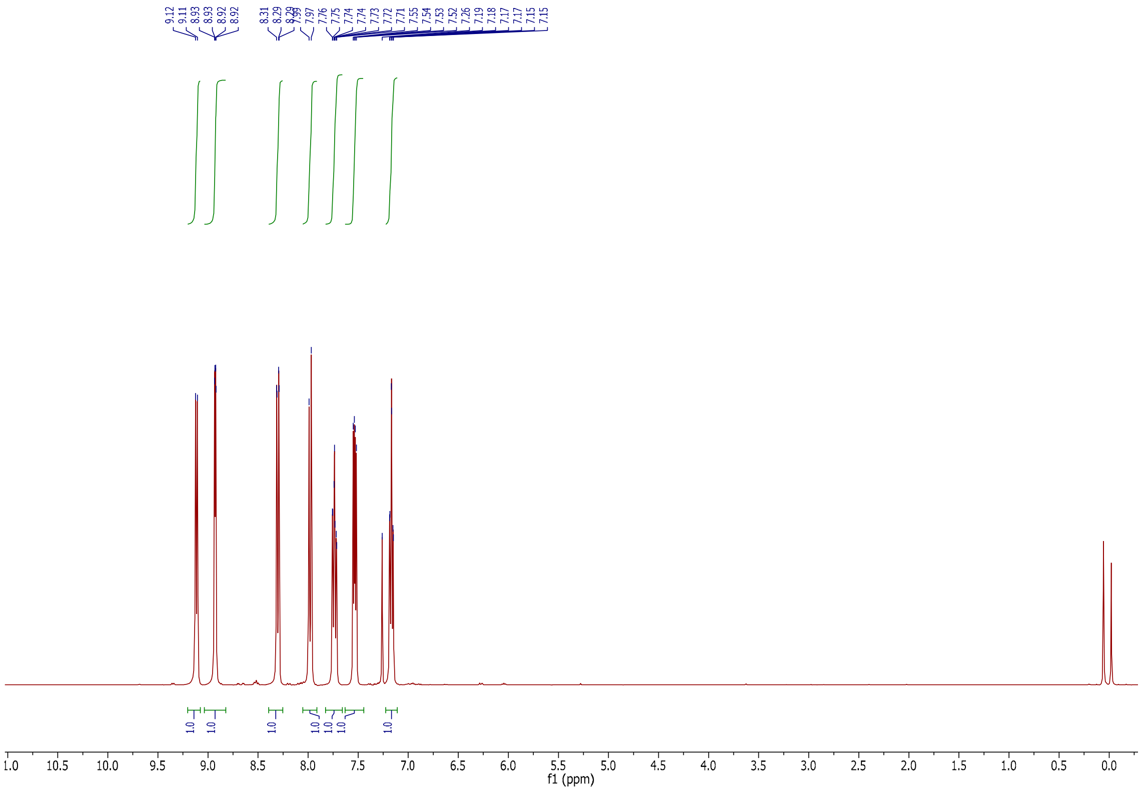

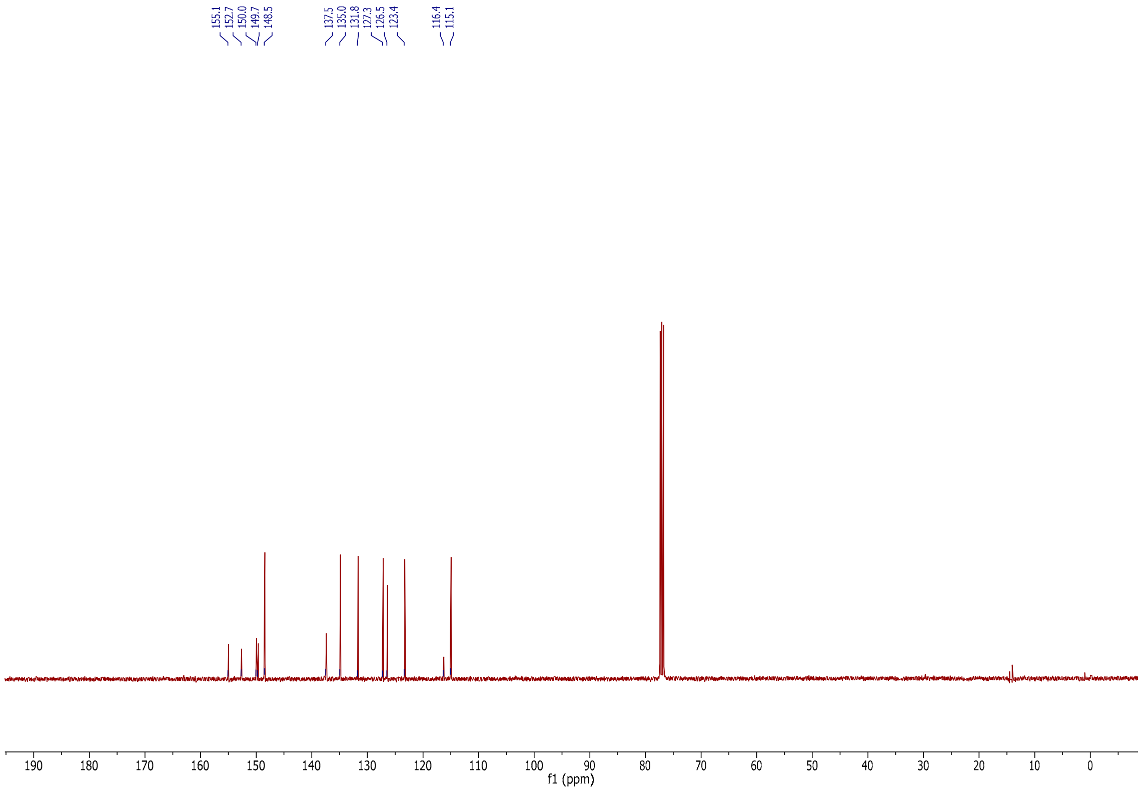

9-fluoro-6*H*-pyrido[1,2-*a*]pyrido[2',3':4,5]thieno[3,2-*d*]pyrimidin-6-one (3)

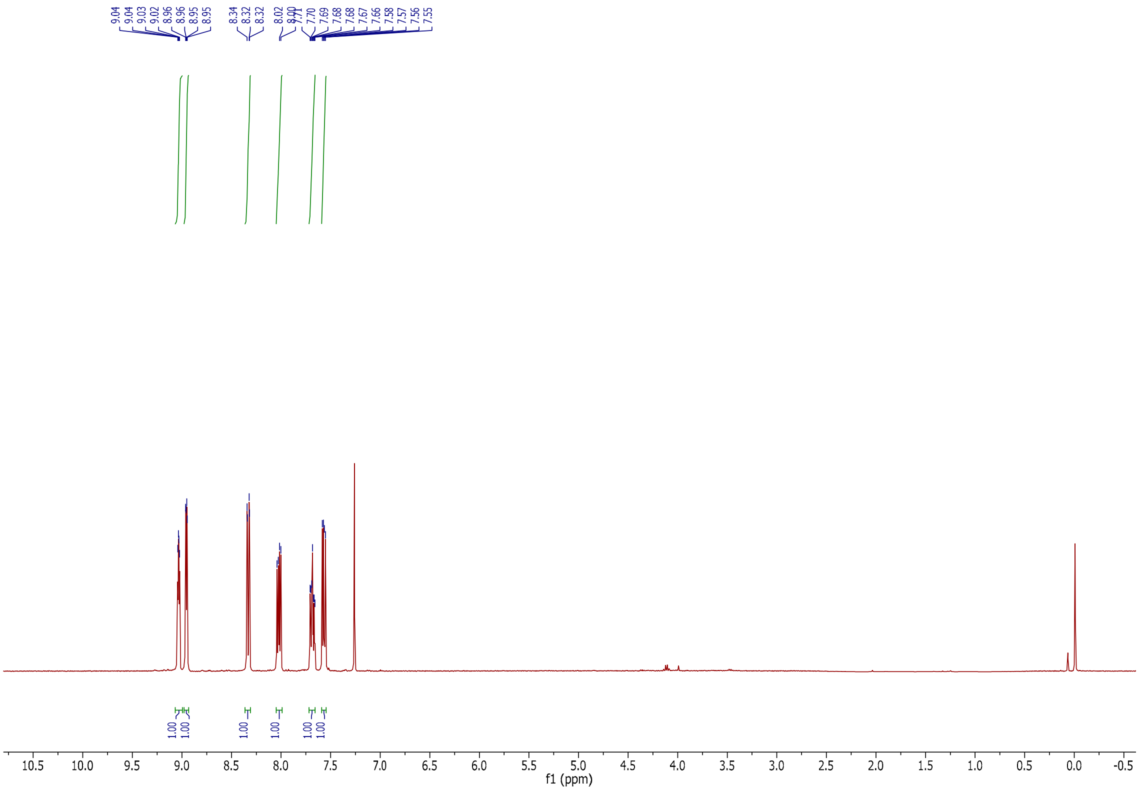

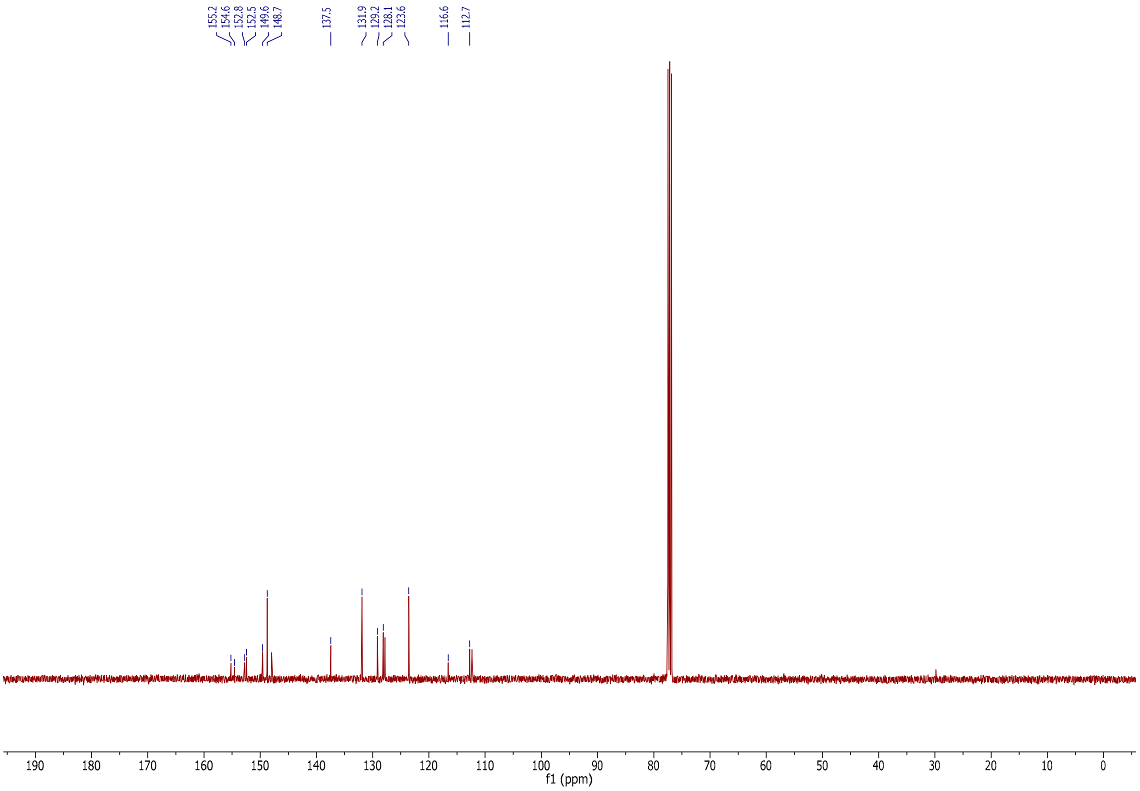

9-chloro-6*H*-pyrido[1,2-*a*]pyrido[2',3':4,5]thieno[3,2-*d*]pyrimidin-6-one (4)

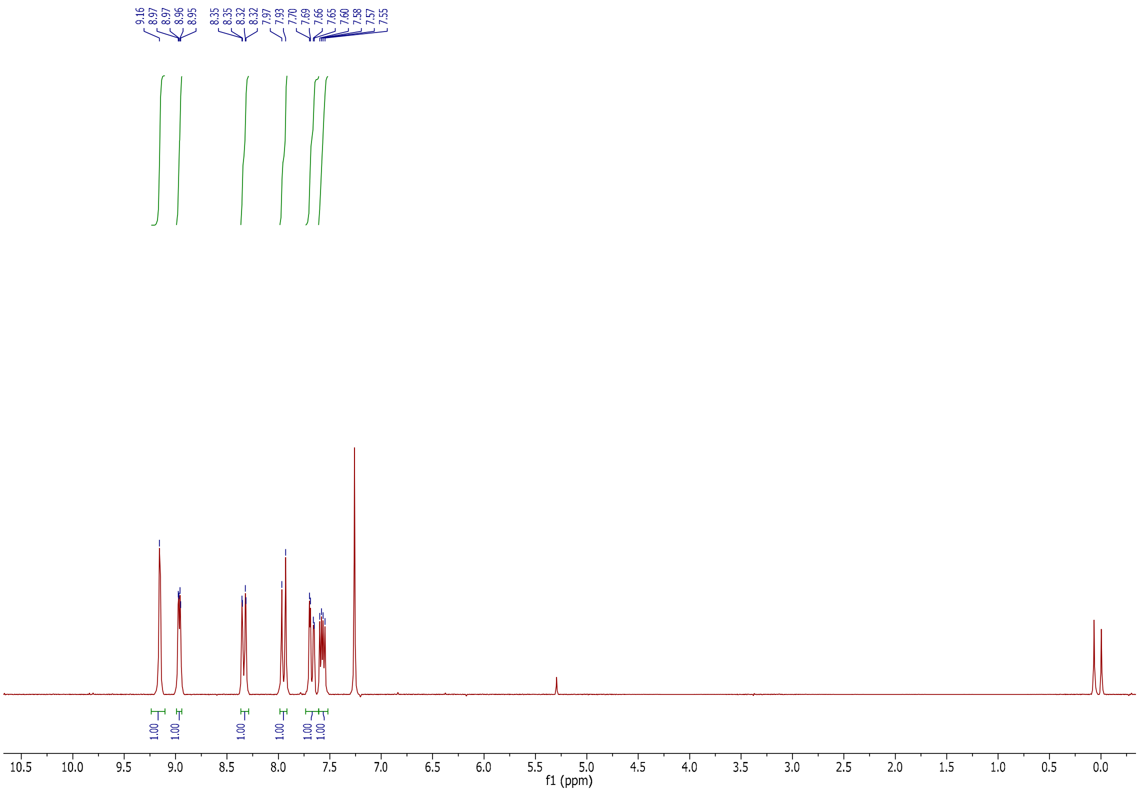

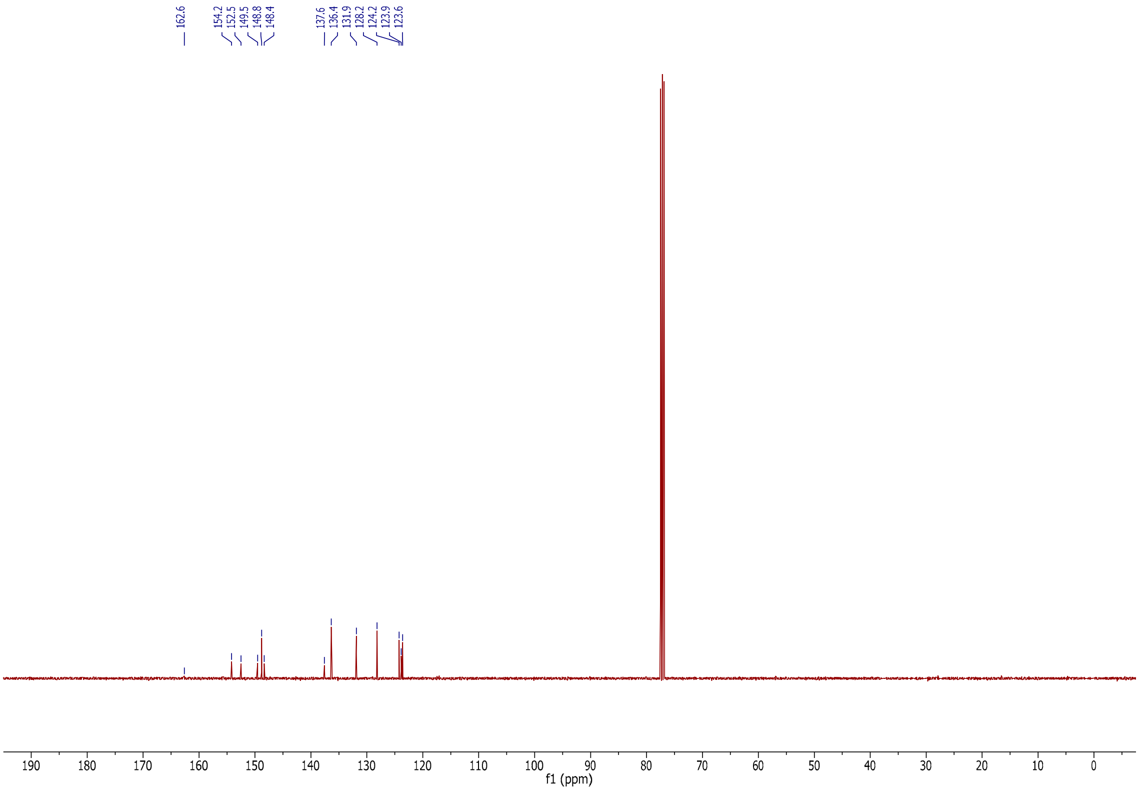

9-methoxy-6*H*-pyrido[1,2-*a*]pyrido[2',3':4,5]thieno[3,2-*d*]pyrimidin-6-one (5)

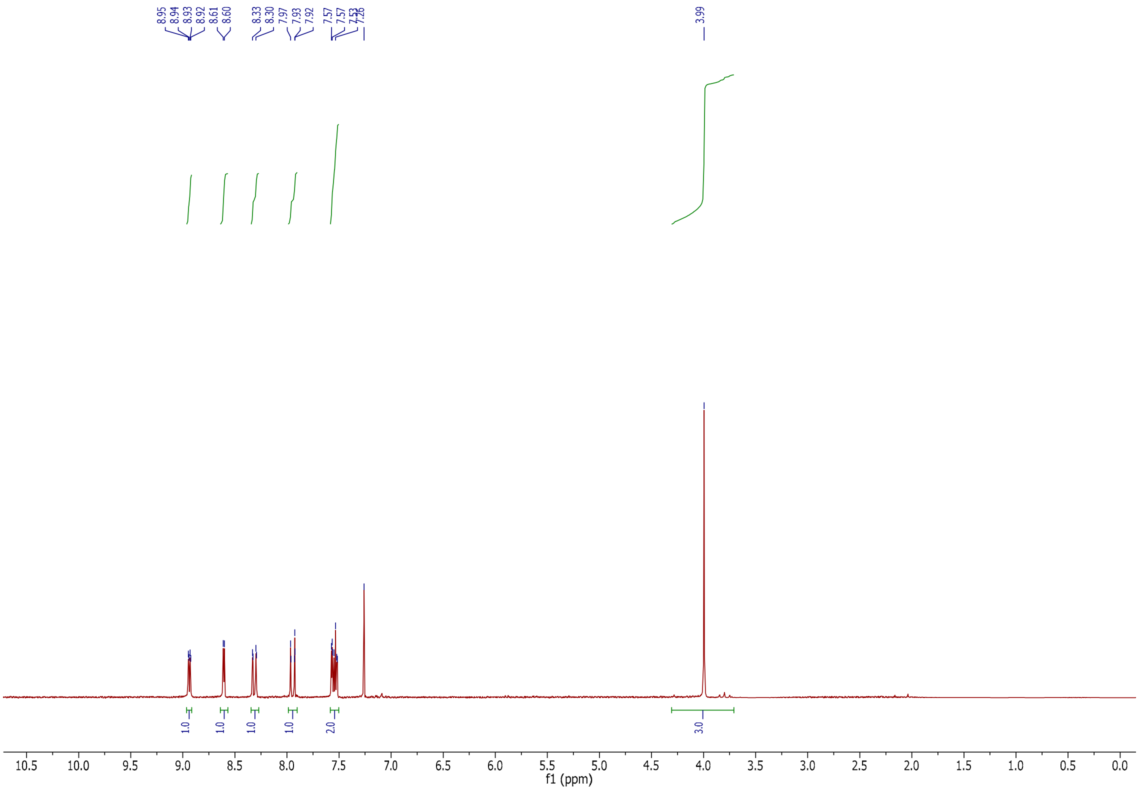

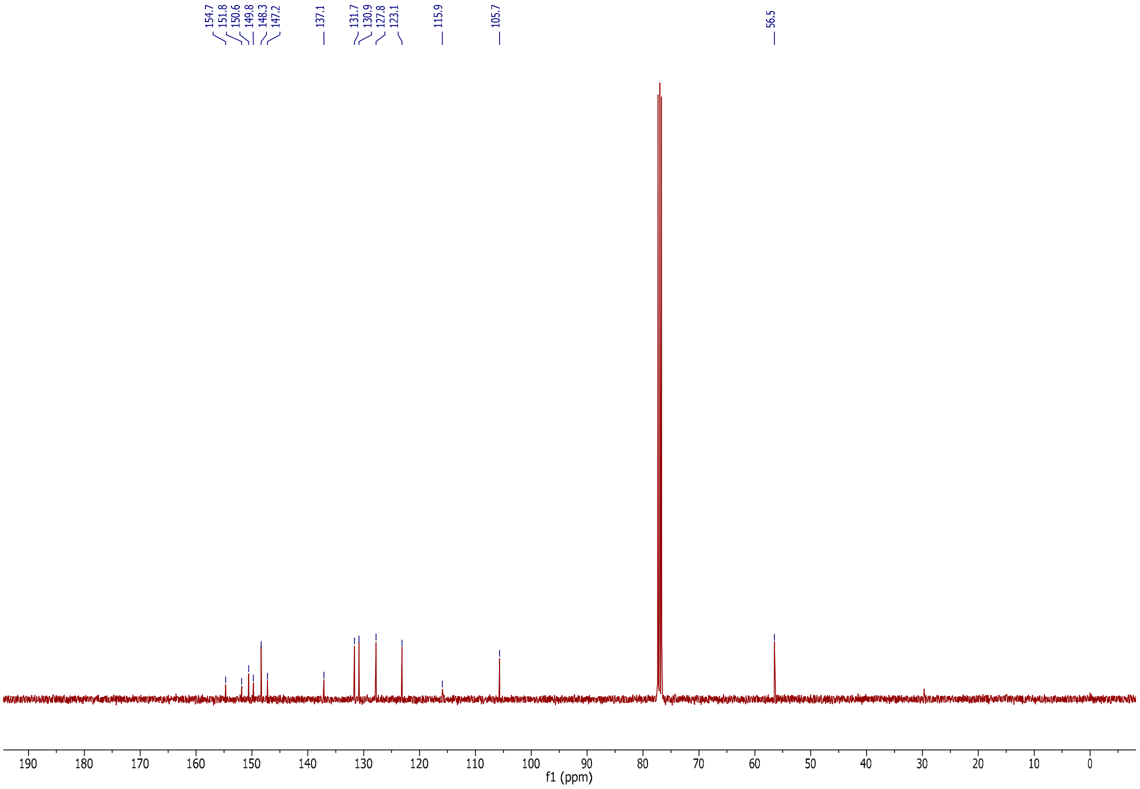

11-methoxy-6*H*-pyrido[1,2-*a*]pyrido[2',3':4,5]thieno[3,2-*d*]pyrimidin-6-one (6)

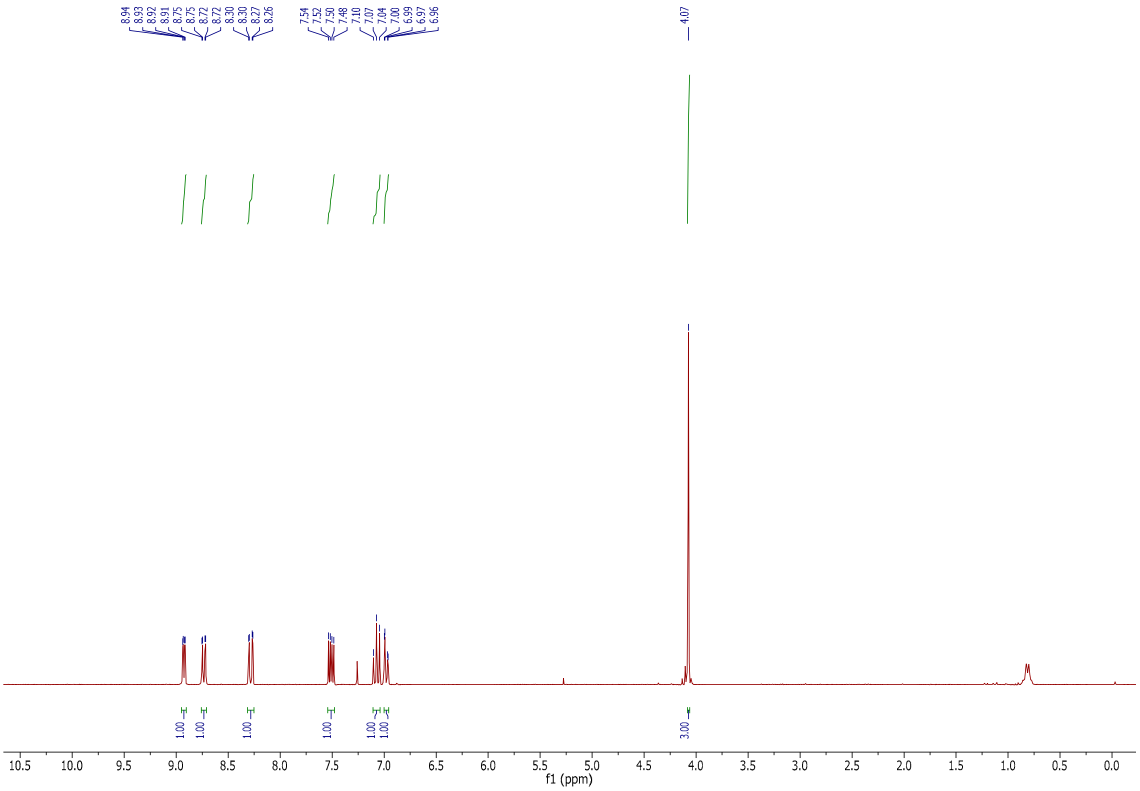

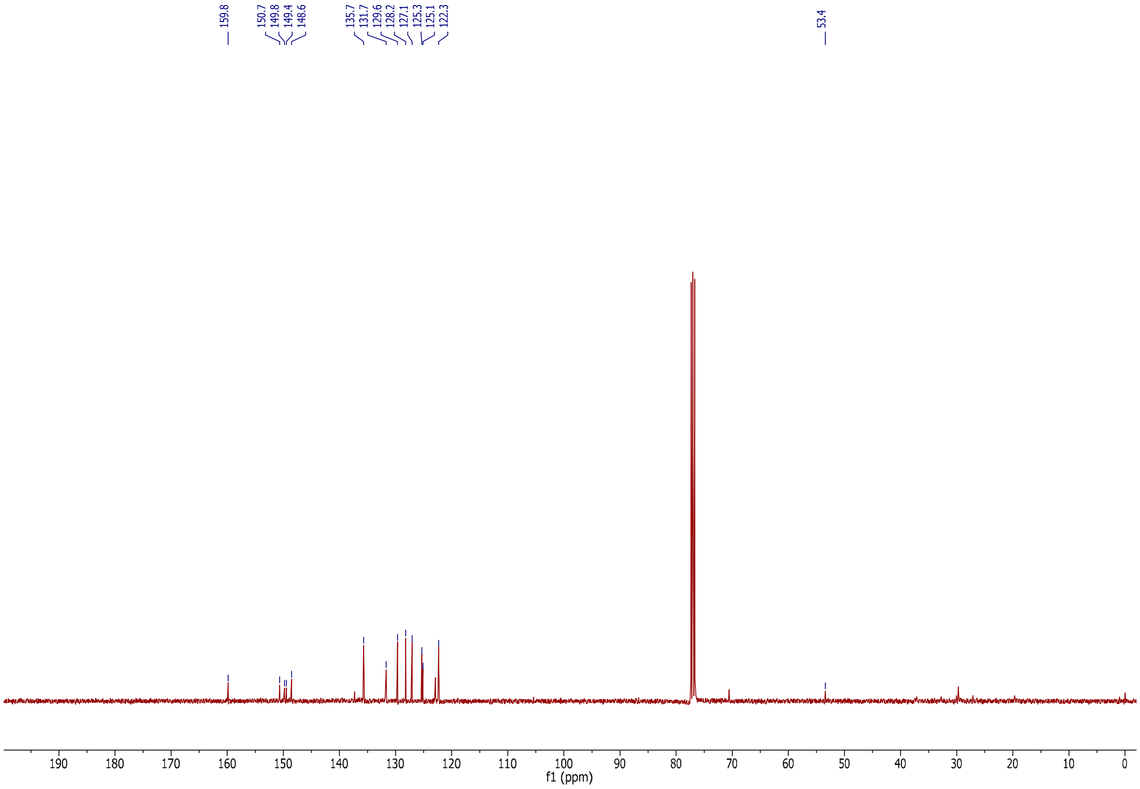

13*H*-pyrido[2'',3'':4',5']thieno[3',2':4,5]pyrimido[1,2-*a*]quinolin-13-one (7)

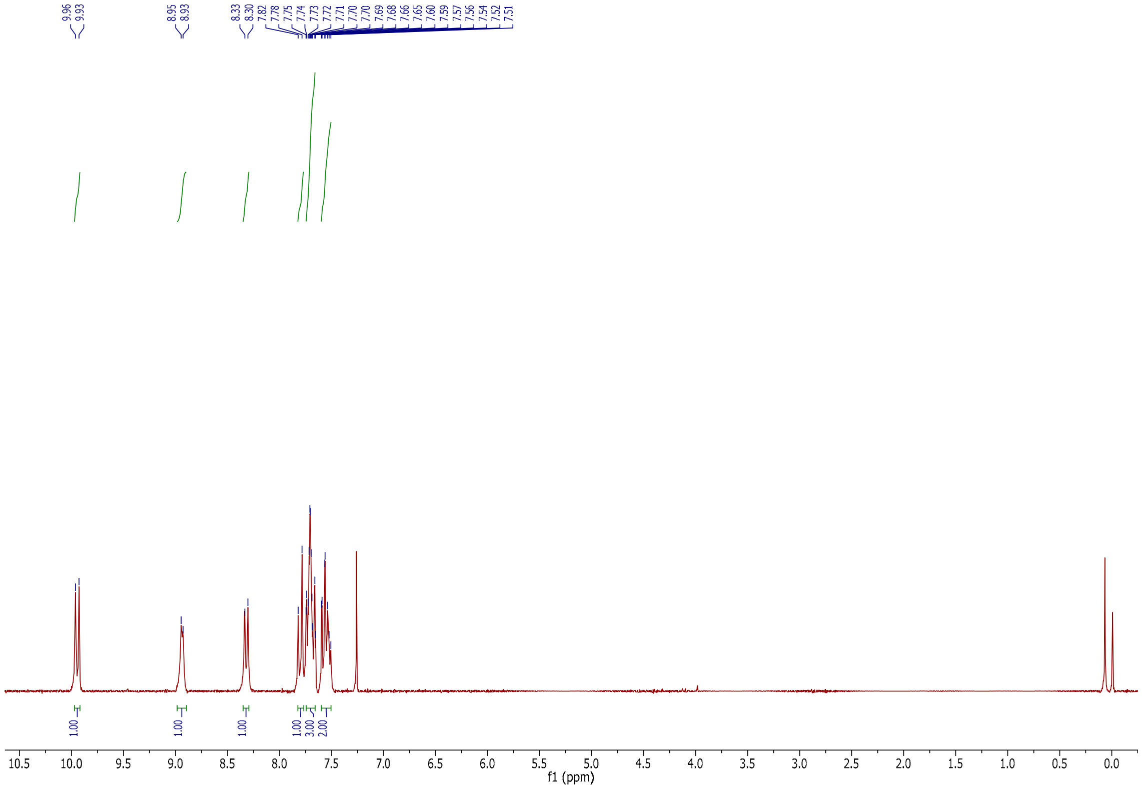

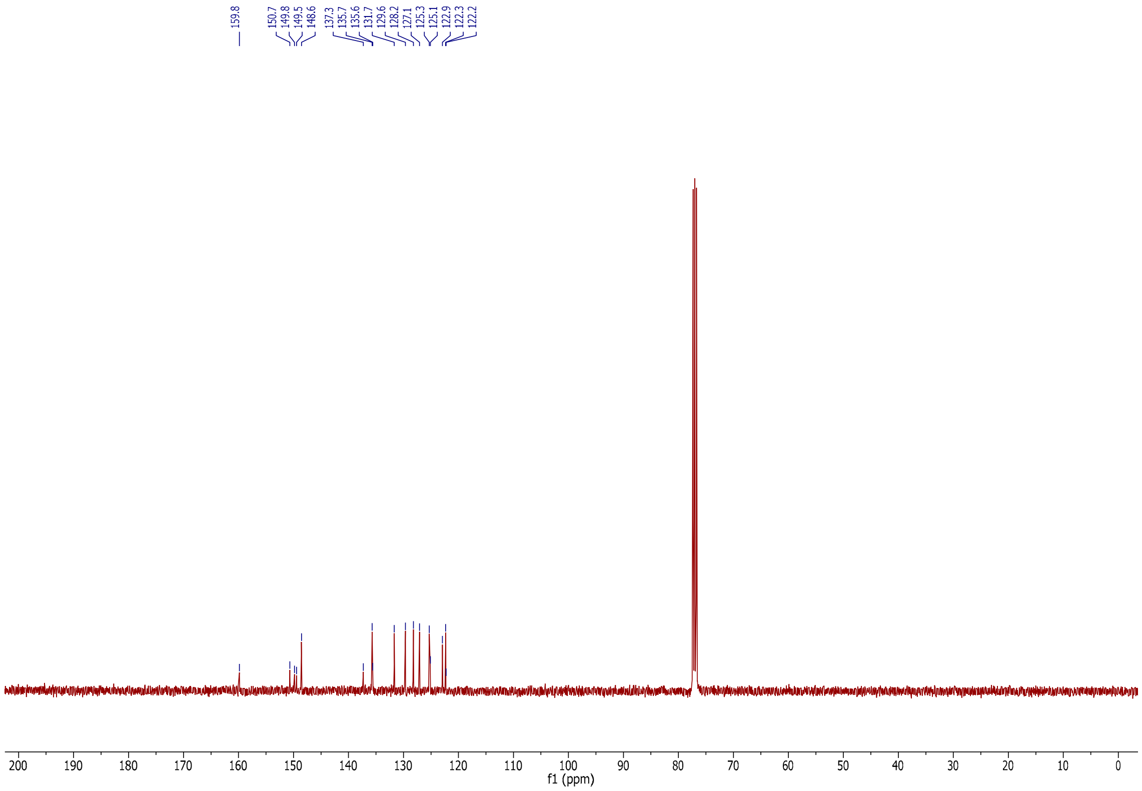

9-methyl-6*H*-pyrido[1,2-*a*]pyrido[2',3':4,5]thieno[3,2-*d*]pyrimidin-6-one (8)

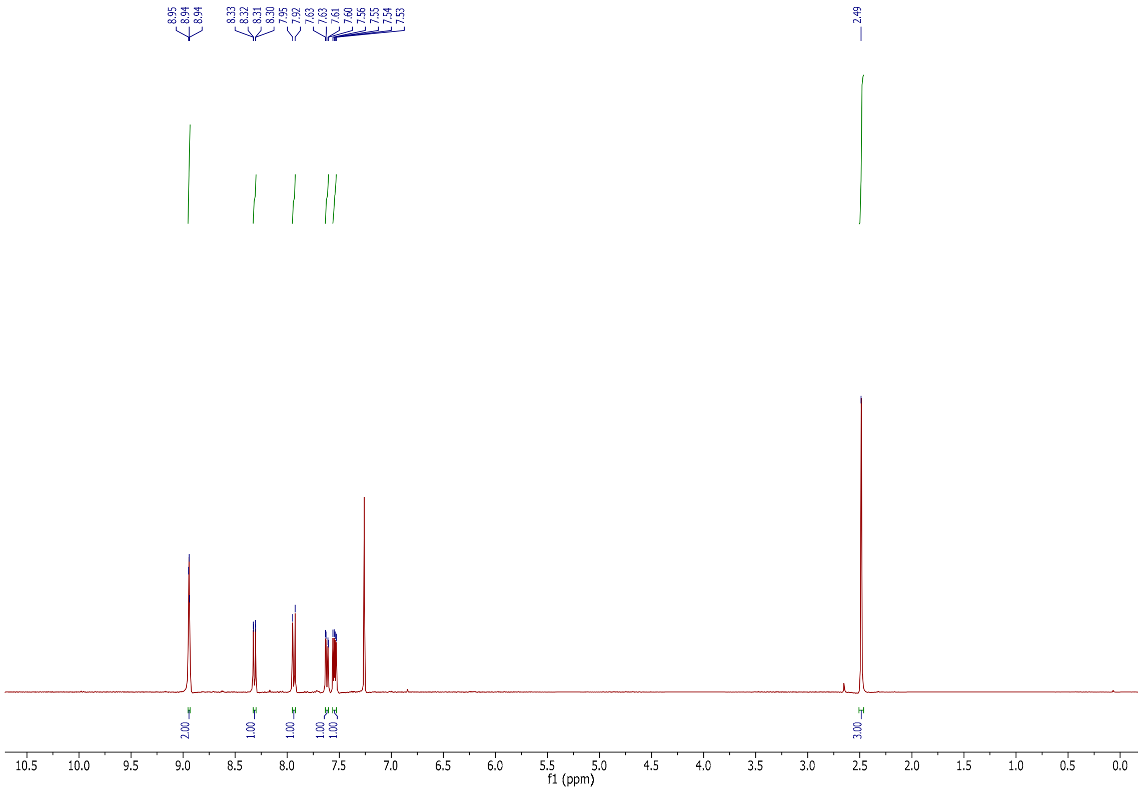

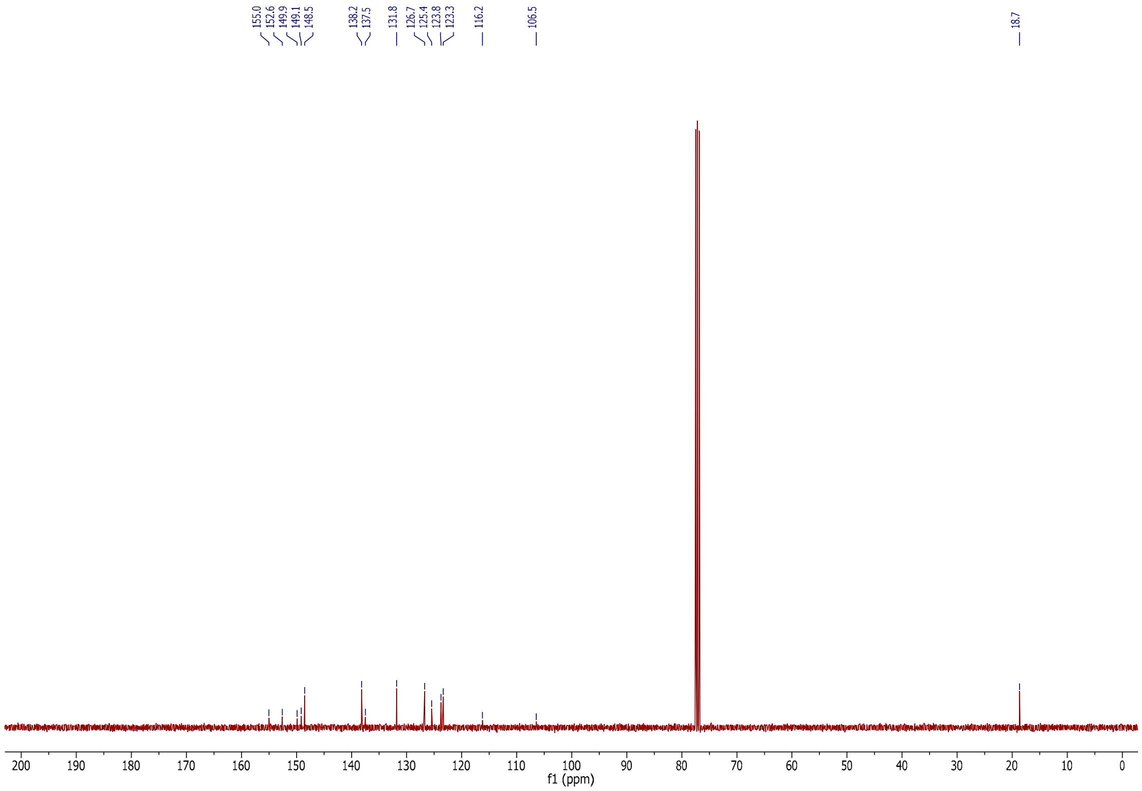

6*H*-pyrazino[2',3':4,5]thieno[3,2-*d*]pyrido[1,2-*a*]pyrimidin-6-one (10)

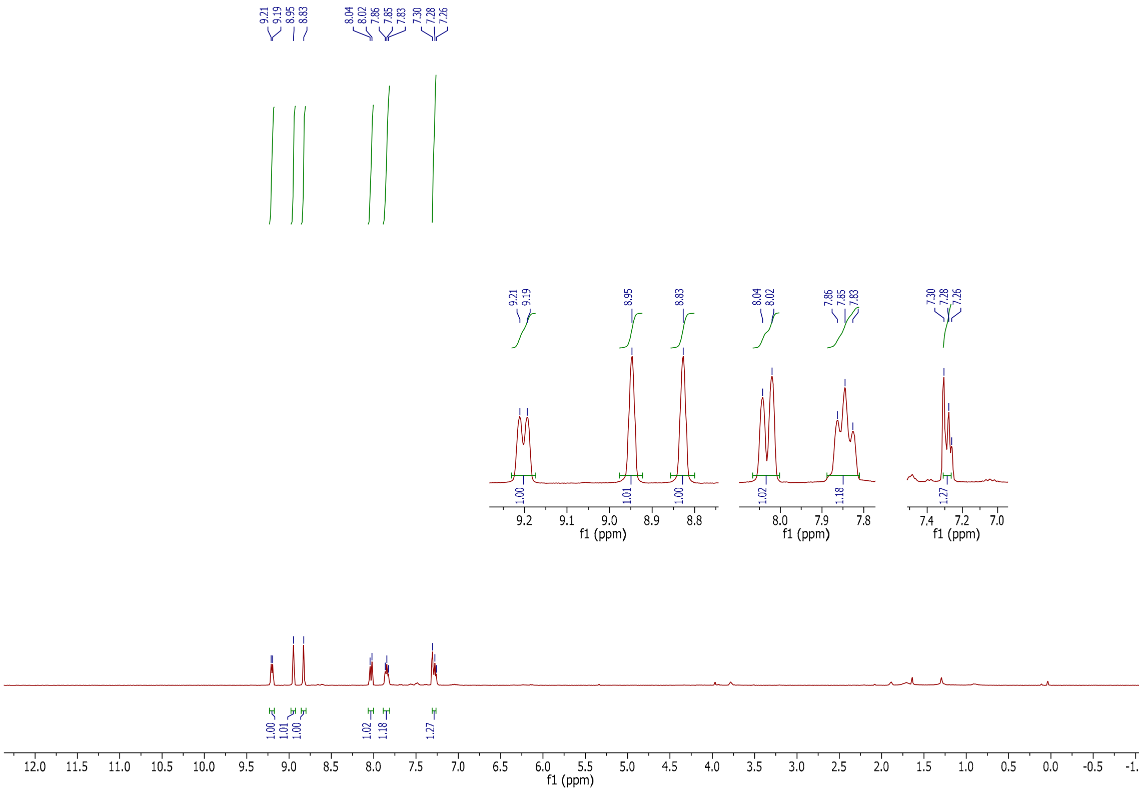

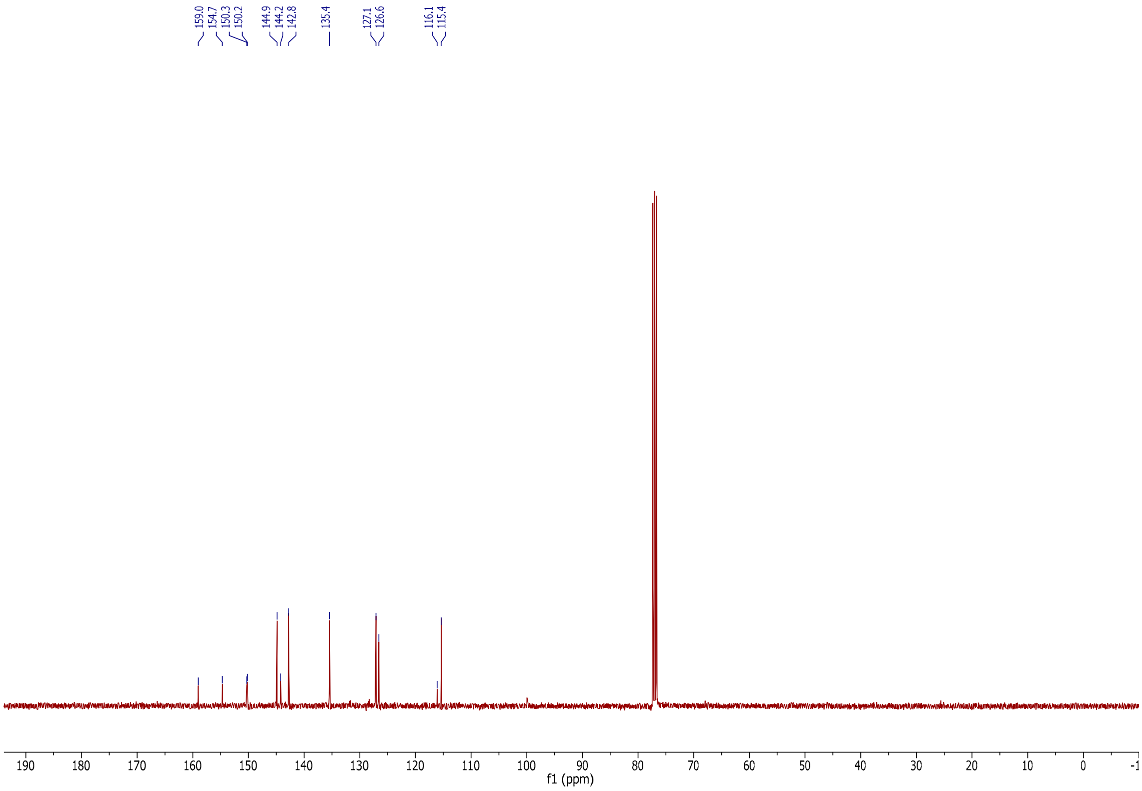

13*H*-pyrazino[2'',3'':4',5']thieno[3',2':4,5]pyrimido[1,2-*a*]quinolin-13-one (11)

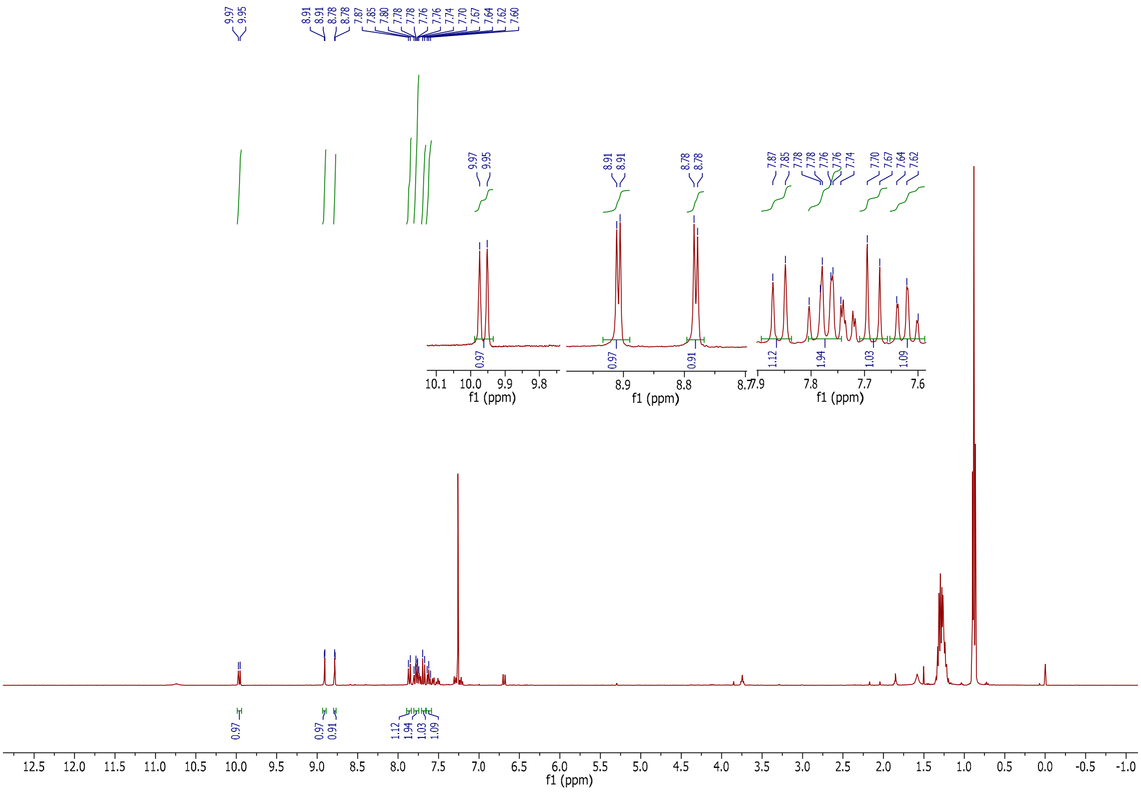

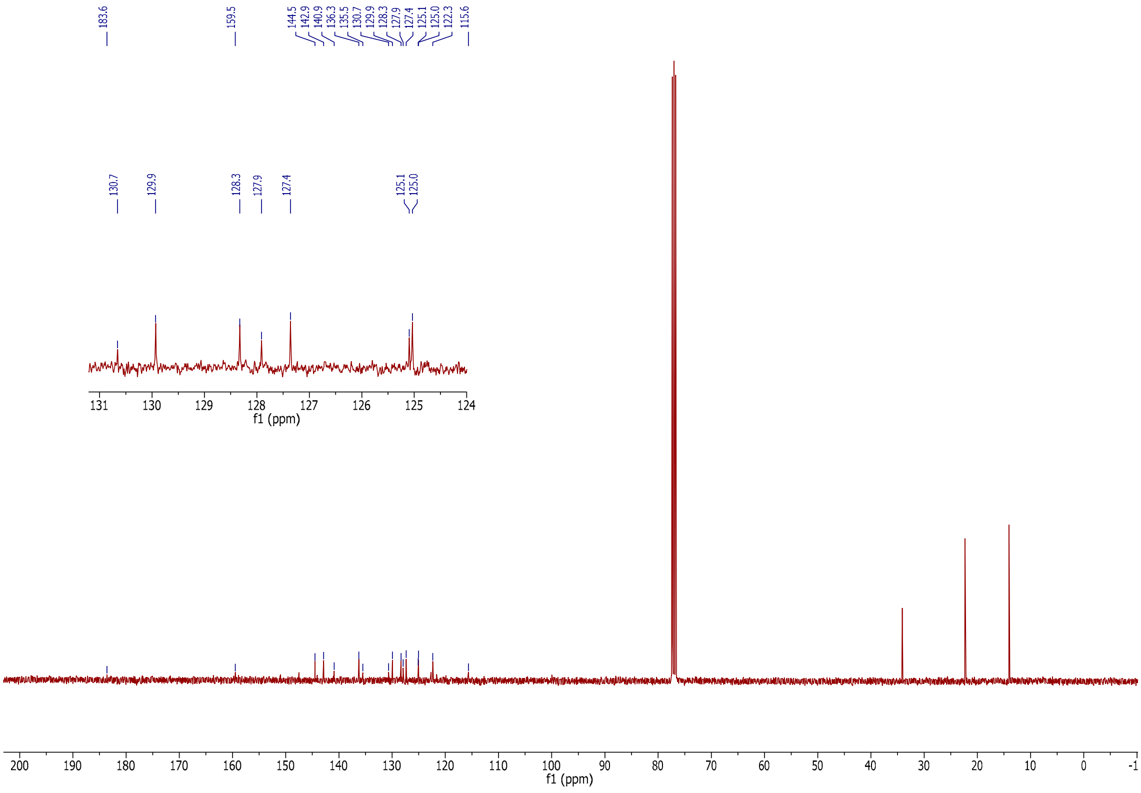

9-fluoro-6*H*-pyrazino[2',3':4,5]thieno[3,2-*d*]pyrido[1,2-*a*]pyrimidin-6-one (12)

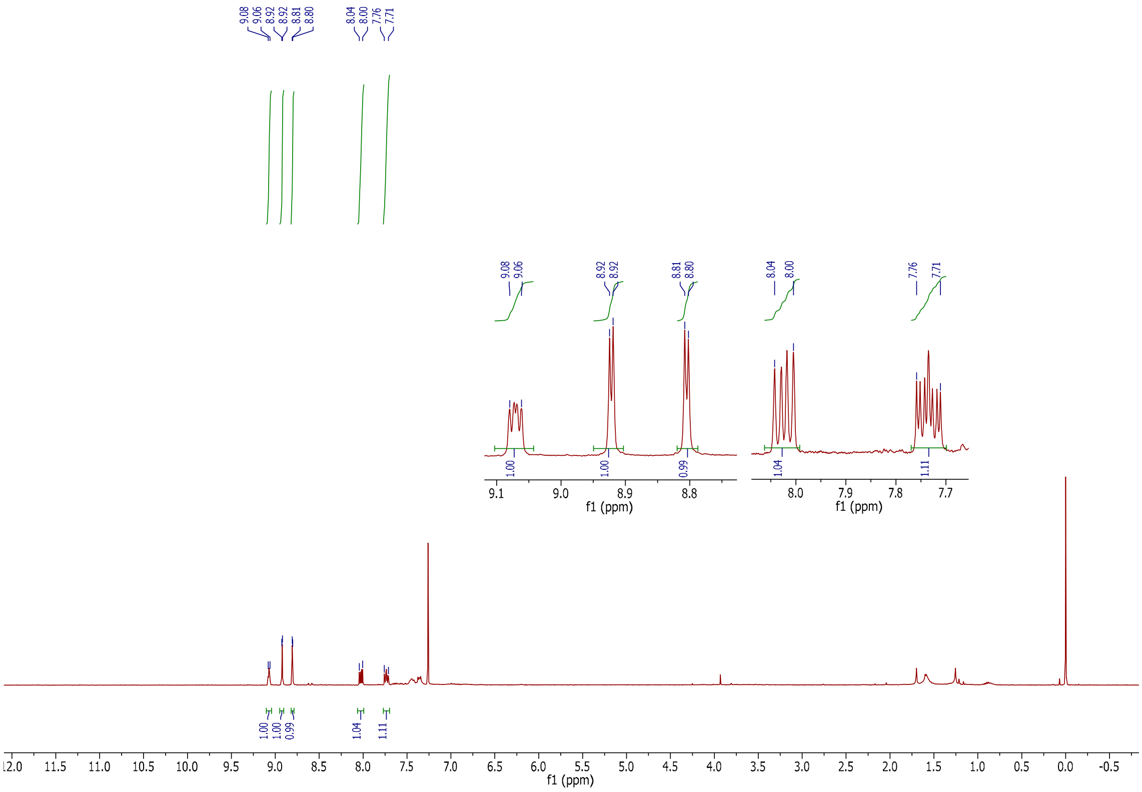

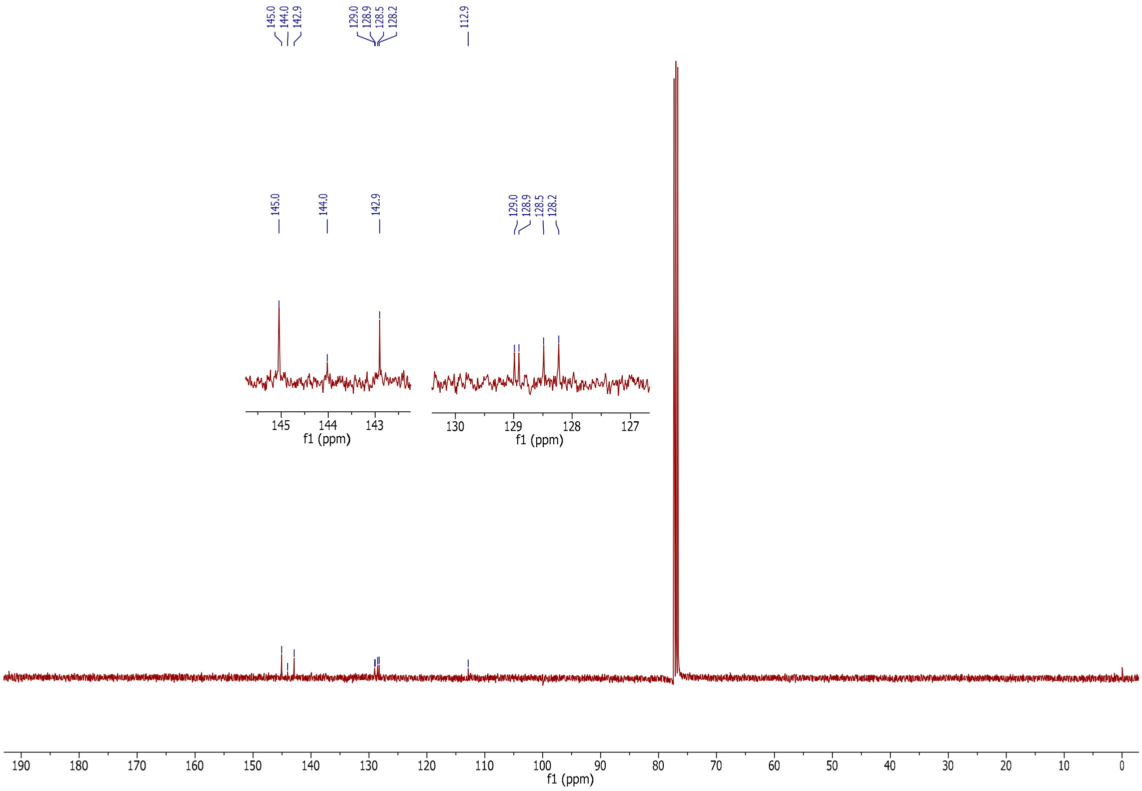

9-chloro-6*H*-pyrazino[2',3':4,5]thieno[3,2-*d*]pyrido[1,2-*a*]pyrimidin-6-one (13)

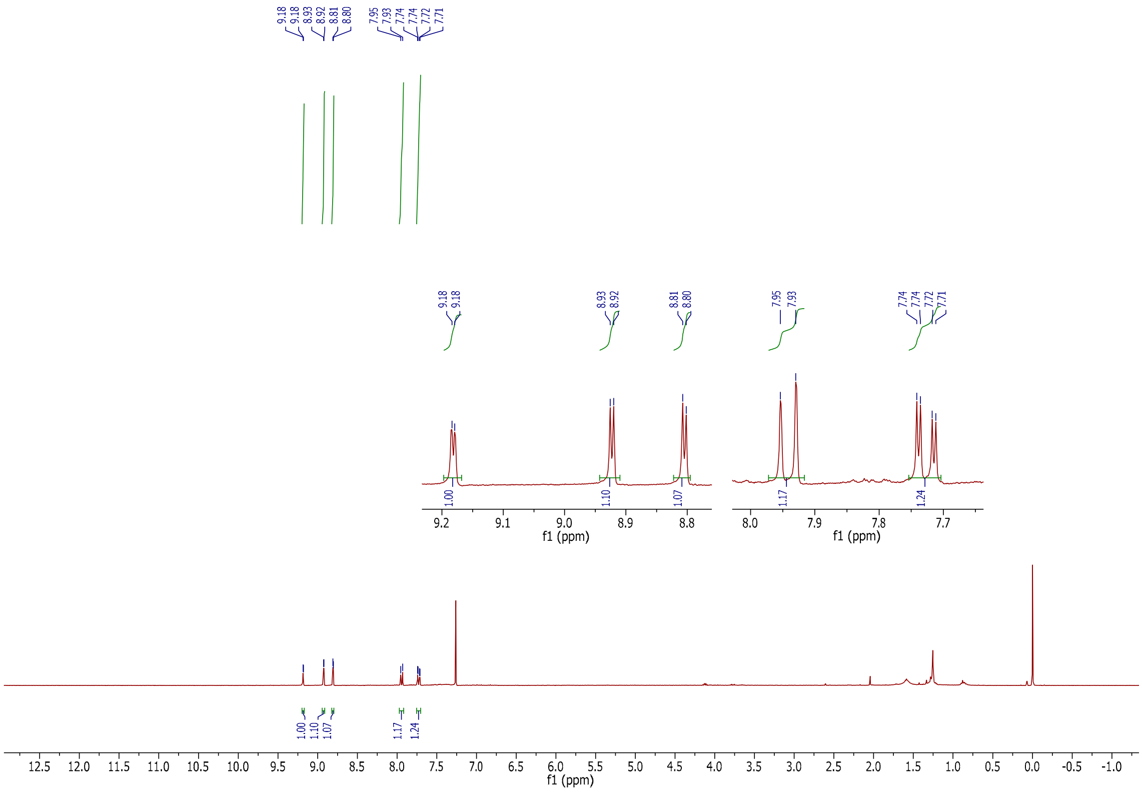

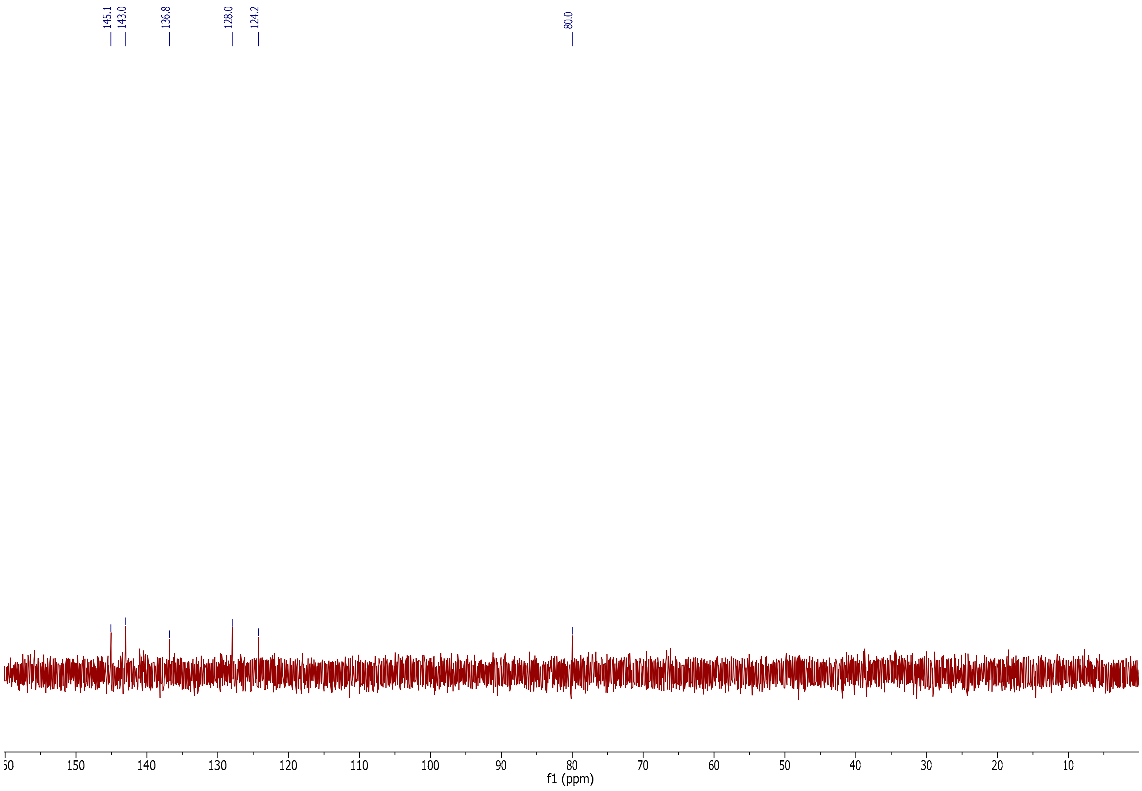

9-(*p*-tolyl)-6*H*-pyrido[1,2-*a*]pyrido[2',3':4,5]thieno[3,2-*d*]pyrimidin-6-one (14)

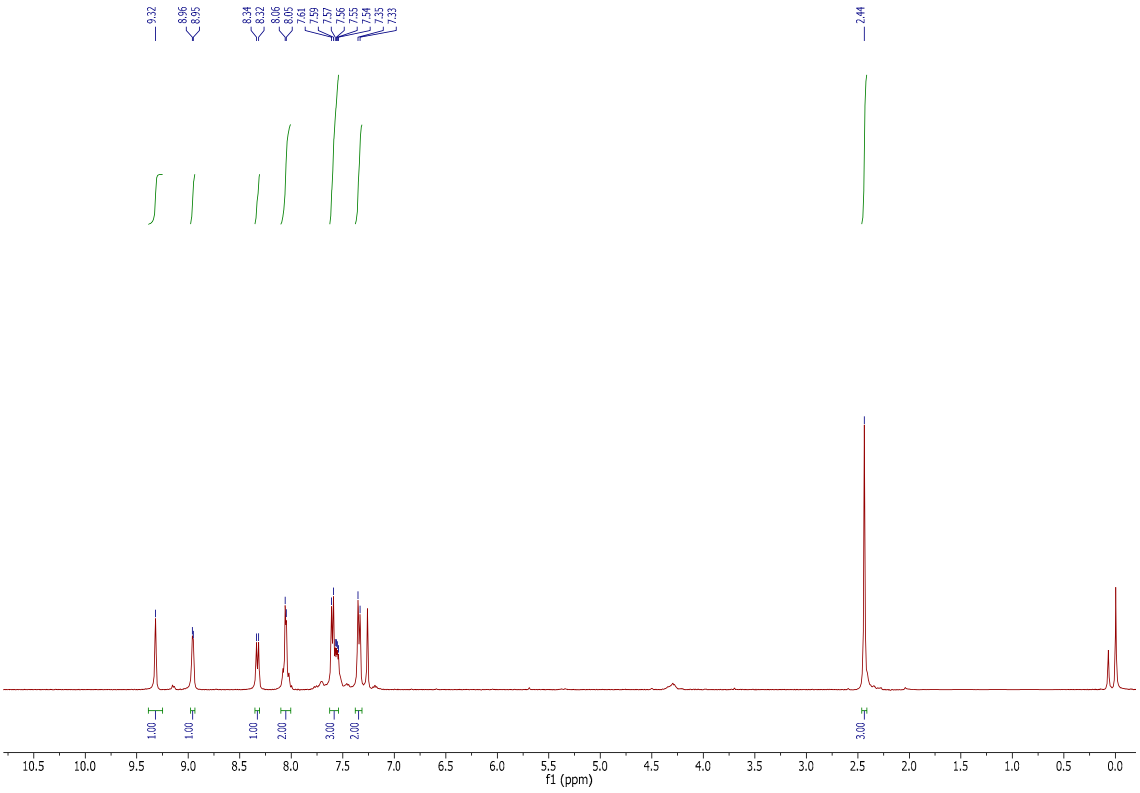

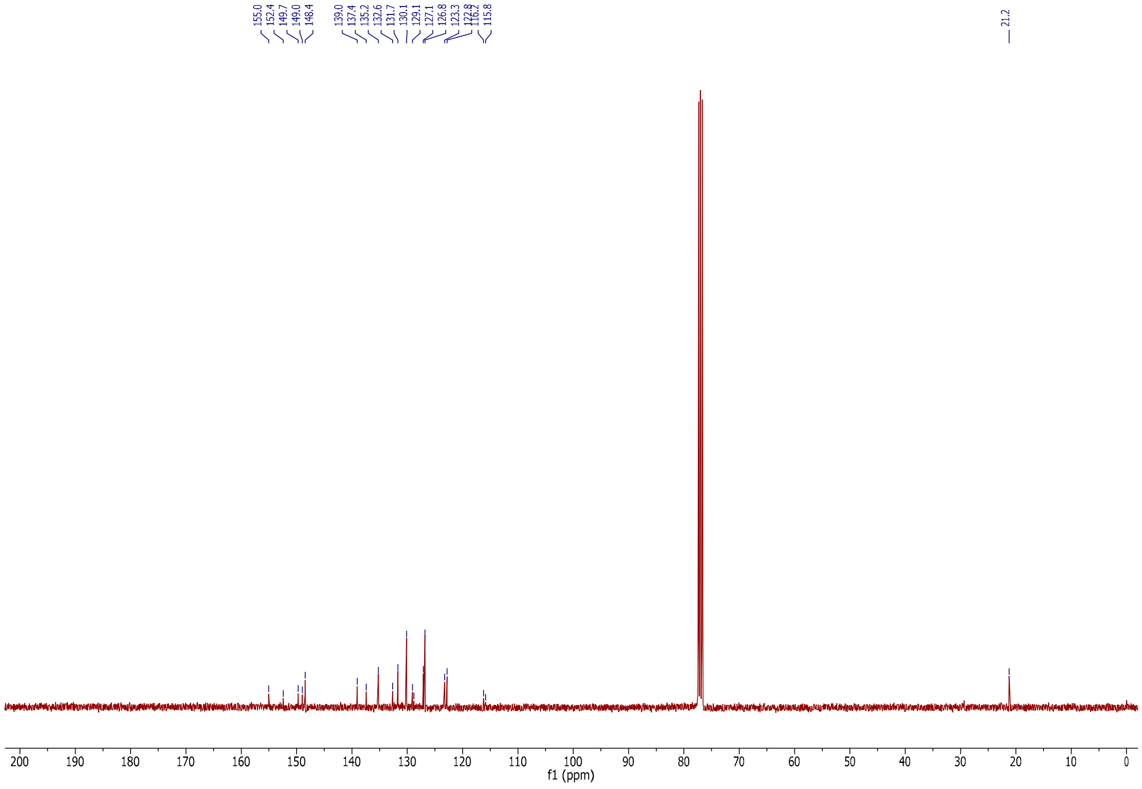

9-(4-methoxyphenyl)-6*H*-pyrido[1,2-*a*]pyrido[2',3':4,5]thieno[3,2-*d*]pyrimidin-6-one (15)

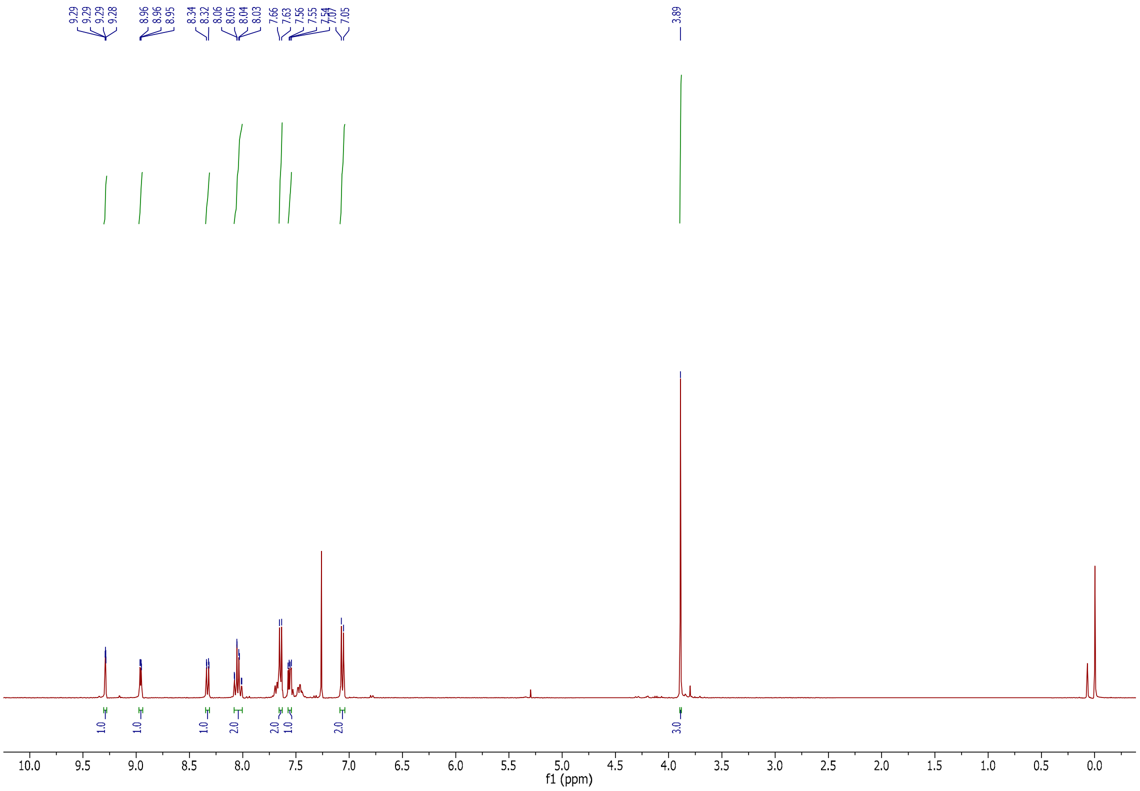

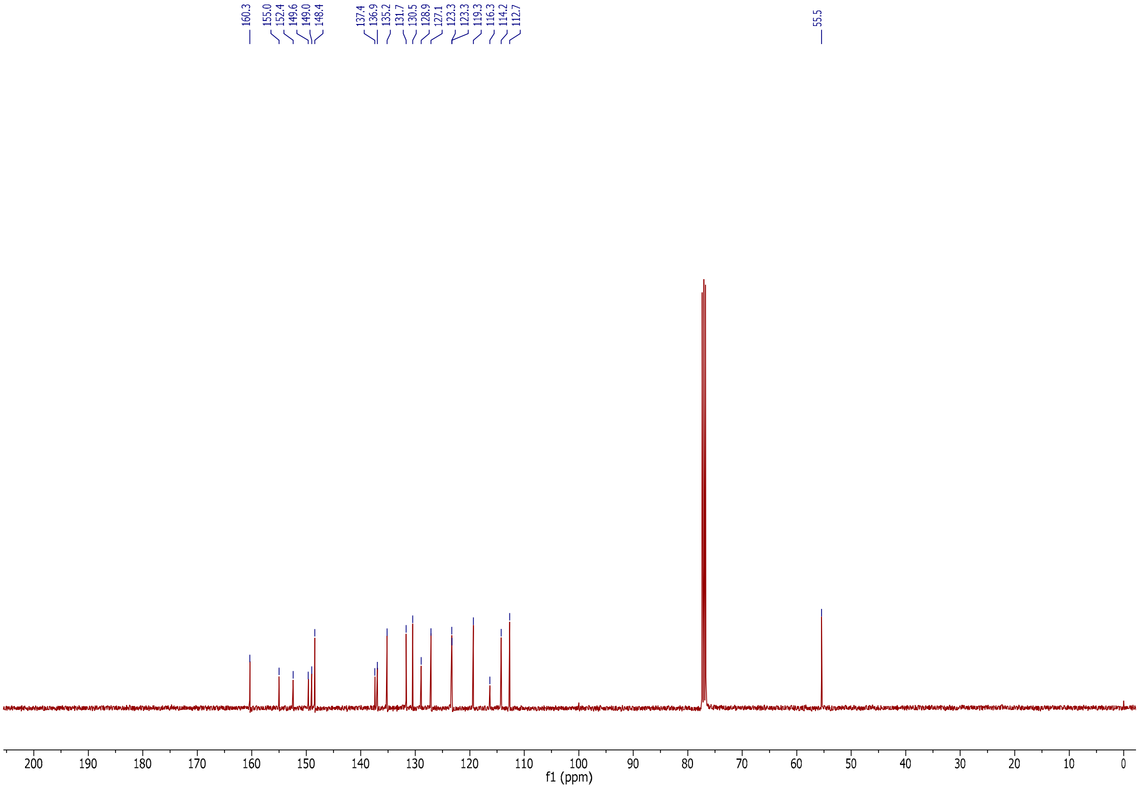

9-(3-methoxyphenyl)-6*H*-pyrido[1,2-*a*]pyrido[2',3':4,5]thieno[3,2-*d*]pyrimidin-6-one (16)

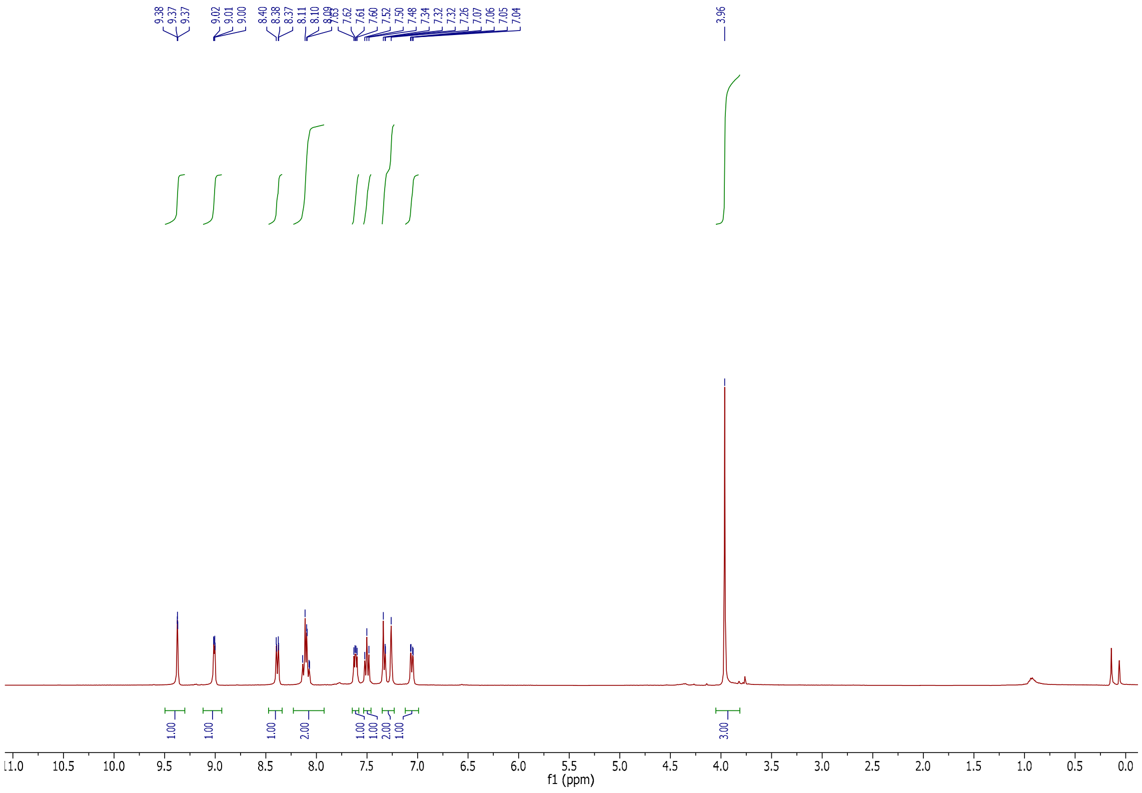

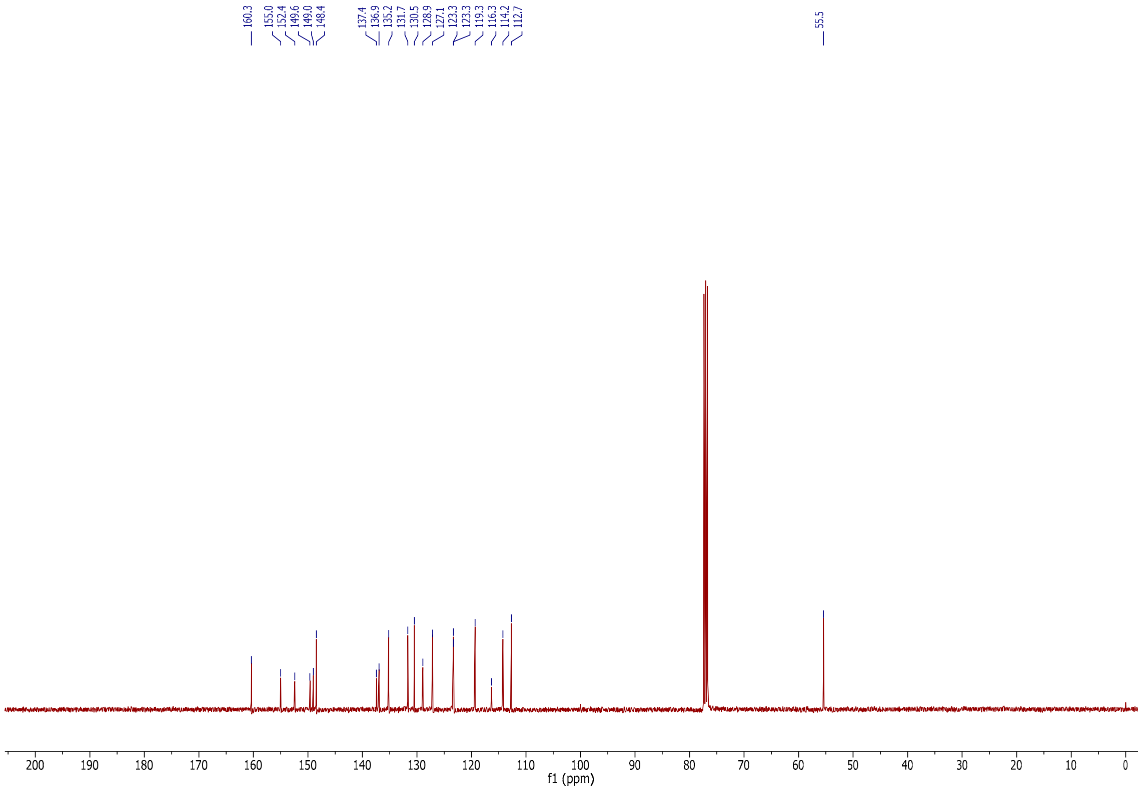

9-(3-(trifluoromethyl)phenyl)-6*H*-pyrido[1,2-*a*]pyrido[2',3':4,5]thieno[3,2-*d*]pyrimidin-6-one (17)

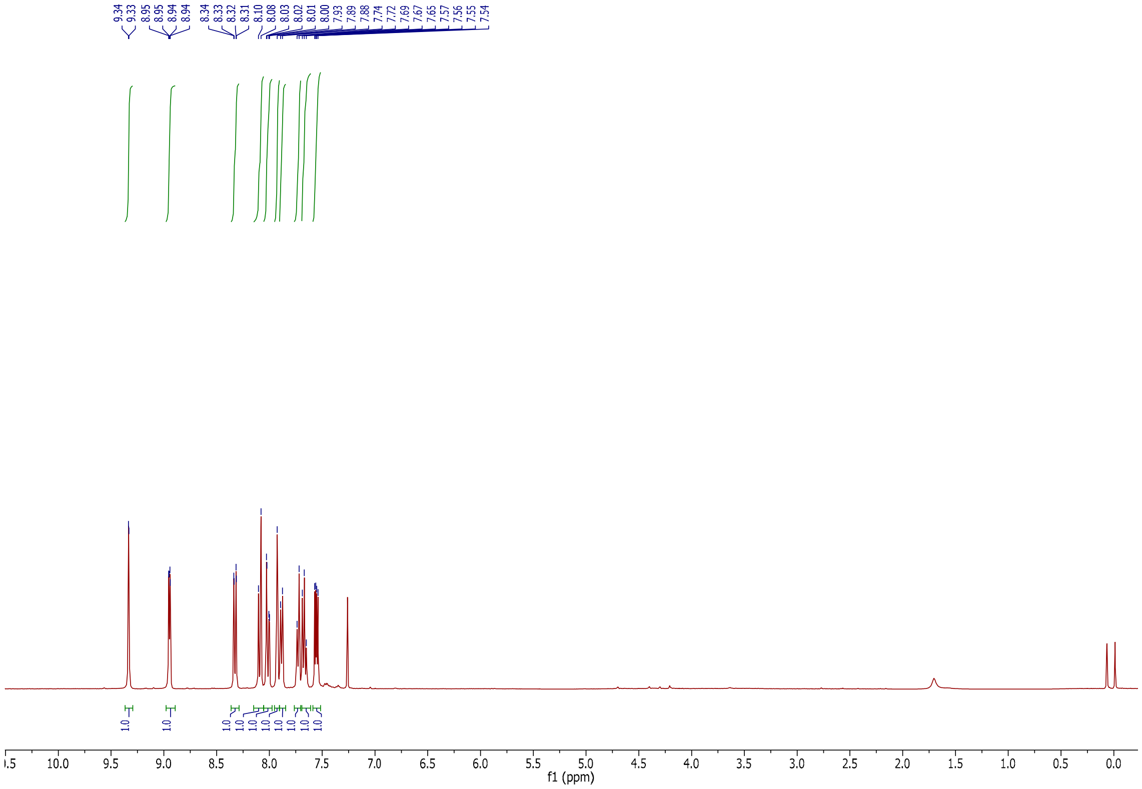

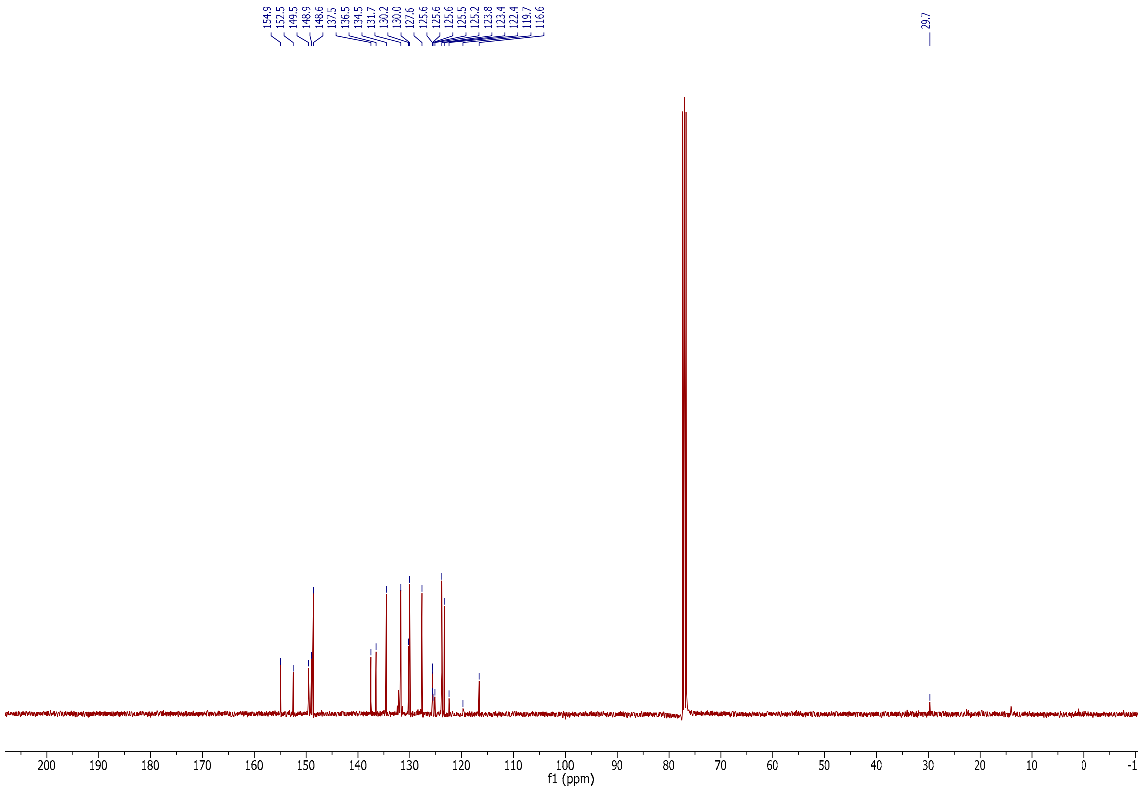

9-(4-(trifluoromethoxy)phenyl)-6*H*-pyrido[1,2-*a*]pyrido[2',3':4,5]thieno[3,2-*d*]pyrimidin-6-one (18)

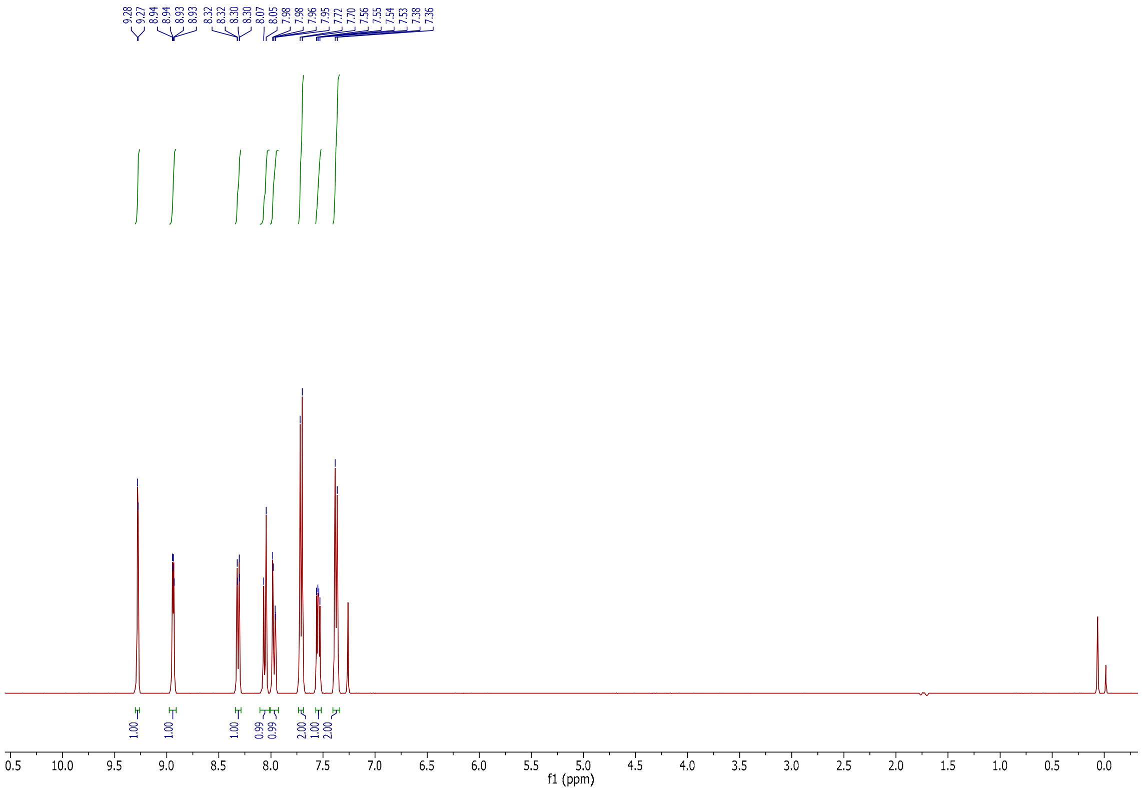

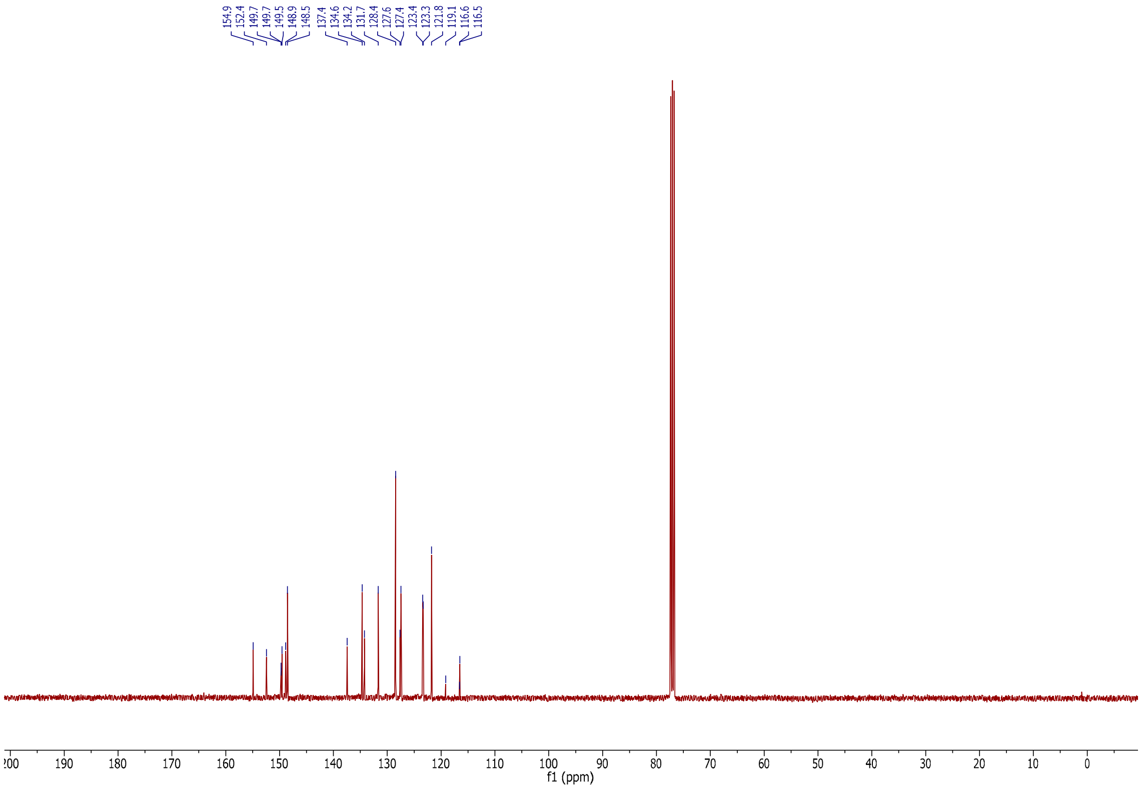

© 2018 by the authors. Licensee MDPI, Basel, Switzerland. This article is an open access article distributed under the terms and conditions of the Creative Commons Attribution (CC BY) license (http://creativecommons.org/licenses/by/4.0/).
